# Potato (*Solanum tuberosum* L., cv. Spunta) Peels Promote the Production of Broad Spectrum Antibacterial Chemicals by *Bacillus velezensis* B38

**DOI:** 10.3390/molecules31162747

**Published:** 2026-08-07

**Authors:** Malek Gharbi, Dorra Gharbi, Ines Karkouch, Abel M. Forero, Jaime Rodríguez, Manel Chaouachi, Takwa Marzouk, Asma Bachali, Carlos Jiménez, Olfa Tabbene

**Affiliations:** 1 Faculty of Sciences of Tunis, University of Tunis El Manar, Tunis 2092, Tunisia; malek.gharbi@cbbc.rnrt.tn; 2 Laboratoire des Substances Bioactives, Centre de Biotechnologie de Borj Cedria, BP 901, Hammam Lif 2050, Tunisia; dorra.gharbi@cbbc.rnrt.tn (D.G.); ines.karkouch@cbbc.rnrt.tn (I.K.); manel.chaouachi@cbbc.rnrt.tn (M.C.); takwa.marzouk@cbbc.rnrt.tn (T.M.); 3 Centro Interdisciplinar de Química e Bioloxía (CICA) and Departamento de Química, Facultade de Ciencias, Universidade da Coruña, 15071 Coruña, Spain; mateo.forerot@udc.es (A.M.F.); jaime.rodriguez@udc.es (J.R.); carlos.jimenez@udc.es (C.J.); 4 Laboratory of Clinical Biochemistry, Mohamed Taher Maamouri Hospital, Nabeul 8000, Tunisia; asma.bachali@fmt.utm.tn

**Keywords:** agro-industrial waste valorization, potato peel waste, *Bacillus velezensis* B38, circular bioeconomy, lipopeptides, oxydifficidin, molecular docking

## Abstract

Potato peel waste (PPW) represents a low-cost and eco-friendly by-product with potential for use as a fermentation substrate for the production of antibacterial metabolites by *Bacillus* species. This study evaluated PPW as a fermentation matrix for *Bacillus velezensis* and demonstrated that 2% PPW supported optimal production of antibacterial compounds. The cell-free supernatant obtained after 96 h of fermentation exhibited the strongest activity against *E. coli* 18/22, with a MIC of 62.5 µg/mL. The ethyl acetate extract exhibited rapid bactericidal activity, killing *E. coli* 18/22 within 30 min at 2× MIC and within 120 min at MIC. The antibacterial activity remained stable up to 80 °C, across pH 4–12, and after treatment with pepsin and chymotrypsin. LC/HR-ESI-MS analysis identified oxydifficidin and surfactins C14 and C15 as the main active compounds. Molecular docking revealed that oxydifficidin and surfactin C15 exhibited the strongest binding affinity toward Shiga toxin II, while surfactin C14 displayed a weaker interaction, suggesting the role of acyl chain length in toxin binding. Both oxydifficidin and surfactins interacted with key residues within the catalytic pocket of the AcrB subunit of the AcrAB–TolC multidrug efflux pump, indicating potential inhibition of multidrug resistance mechanisms. To our knowledge, this is the first report describing the use of PPW as a fermentation substrate for *Bacillus velezensis* B38 to produce oxydifficidin and surfactin C14 and C15 exhibiting antimicrobial activity against multidrug-resistant *E. coli*. These findings provide a promising strategy for transforming agro-industrial waste into value-added antimicrobial metabolites, contributing to the circular bioeconomy strategies within the One Health framework.

## 1. Introduction

Potato peel waste (PPW) is one of the main by-products of the potato processing industry, resulting from large-scale production of chips, fries, starch, and other derivatives [1]. Given the global importance of potato as a staple crop, improper management of PPW can lead to environmental and economic challenges. Nevertheless, growing interest has focused on its potential valorization through circular economy and biorefinery approaches, aiming to convert this residue into value-added products [2,3]. PPW contains residual amounts of starch, fibers, polyphenols, minerals, and vitamins, making it suitable as a low-cost substrate for microbial fermentation process [4]. Accordingly, it has been explored for the production of enzymes [5], bioethanol [6], and biosurfactants [7]. Biorefinery approaches have been proposed to convert PPW into biofuels, bioplastics, and other biomaterials, contributing to the diversification of renewable resource utilization [8,9]. In parallel, opportunistic pathogens such as *Escherichia coli* (*E. coli*), particularly uropathogenic strains (UPEC) and Shiga toxin-producing *E. coli* (STEC), remain a major public health concern [10,11]. STEC virulence is mainly associated with Shiga toxin production, which induces severe intestinal damage and hemolytic–uremic syndrome [12]. Moreover, *E. coli* possesses remarkable resistance to antimicrobial agents, attributed to the AcrAB–TolC multidrug efflux pump, one of the most prominent resistance mechanisms in Gram-negative bacteria. This tripartite pump actively expels a broad spectrum of antibiotics including fluoroquinolones, β-lactams, aminoglycosides, tetracyclines, and macrolides, making AcrAB–TolC efflux pump a key determinant of multi-drug-resistant phenotype of *E. coli* [13]. The widespread occurrence of multidrug-resistant *E. coli* strains highlights the urgent need for sustainable natural antimicrobial alternatives. *Bacillus* species are well known producers of antimicrobial secondary metabolites, including cyclic lipopeptides such as surfactins, fengycins, iturins, and bacillomycins, as well as several polyketides including macrolactin, bacillaene, difficidin and oxydifficidin [14,15]. Among these compounds, surfactins represent a major class of surface-active lipopeptides with broad biological activities [16], while oxydifficidin is a potent polyketide antibiotic reported for its strong antibacterial properties [17]. Their biosynthesis involves non-ribosomal peptide synthetases (NRPSs) for lipopeptides and polyketide synthases (PKSs) for polyketides [18].

Using PPW as a fermentation substrate for *Bacillus* species may offer practical advantages by providing a low-cost alternative to conventional media components while supporting sustainable bioprocess development [3,4,6,19]. Although numerous studies have reported the use of agro-industrial wastes as fermentation substrates for the production of antimicrobial compounds by *Bacillus* species, the potential of *Bacillus velezensis* B38 cultivated on PPW to generate bioactive metabolites active against multidrug-resistant (MDR) *E. coli* remains unexplored. In this context, the present study investigates the use of PPW as a sustainable and low-cost fermentation substrate for *B. velezensis* B38 for the biosynthesis of antibacterial lipopeptide and polyketide metabolites. Special attention was given to the characterization of oxydifficidin and surfactins C14–C15 and their activity against human pathogenic MDR *E. coli* isolates. By integrating agro-industrial waste valorization with the generation of bioactive metabolites, this work highlights a promising strategy for producing value-added antibacterial agents while supporting circular economy and One Health approaches.

## 2. Results and Discussion

### 2.1. Composition of Potato Peel Waste Powder

The compositional analysis of the raw PPW powder is presented in Table S1. PPW exhibited notably ash content (9.389%), indicating a substantial presence of mineral components. This value is comparable to those previously found [20], who reported ash contents ranging between 8.33 and 16.03% in fresh PP powder from four different varieties. The moisture content was also determined to be 15.95 g/100 g DW, which is higher than values previously reported for PPW, ranging from 6.35% to 10.29% [6,21]. Furthermore, the total carbohydrate content of PPW powder was 28.58 g/100 g DW, which is higher than the value reported by Schieber et al. [22] (12.44 g/100 g DW). These results indicate that carbohydrates constitute a major component of PPW, in agreement with previous studies [3,6]. The nitrogen content was determined to be approximately 1.552 g/100 g DW, which aligns with the range reported in the literature between 1.2 and 2.1 g/100 g of nitrogen [23,24]. The total phenolic content (TPC) of PPW was 0.42 g/100 g DW, slightly lower than that reported by [24] who found TPC between 0.51 and 0.96 g/100 g DW. Similarly [25] reported higher phenolic contents across different potato varieties ranging from 0.54 to 3.59 mg GAE g^−1^ DW and up to 12.59 mg GAE g^−1^ DW, depending on the cultivar and extraction conditions [25]. The variation in chemical composition of PPW may be attributed to several factors, including potato cultivar, growth conditions, maturity at harvest, postharvest storage, processing methods, and drying temperature [3].

### 2.2. Molecular Identification of the Bacterium

**Figure 1:**
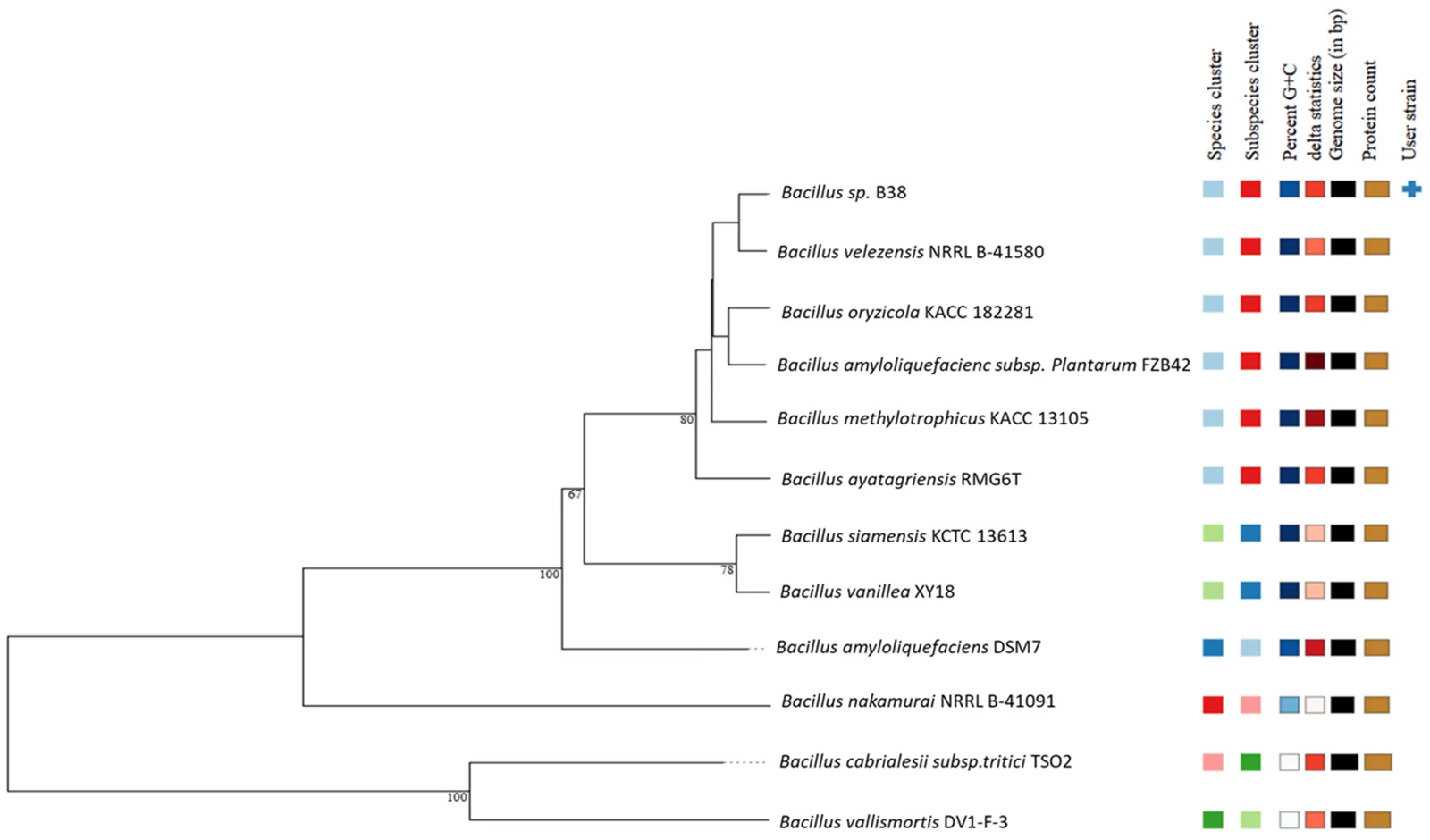
Neighbor joining tree of the studied bacterial strain.

The taxonomic affiliation of *Bacillus* sp. B38 was determined using the Type Strain Genome Server (TYGS) and Average Nucleotide Identity (ANI) analysis via Proksee. Digital DNA–DNA hybridization (dDDH) revealed that strain B38 exhibited the highest similarity to *Bacillus velezensis* NRRL B-41580, with dDDH values of 94.0% (d0), 92.8% (d4), and 95.8% (d6), all well above the 70% species delineation threshold. ANI analysis against *B. velezensis* FZB42 (GCF_000015785.2) yielded a value of 98.05%, with 1202 orthologous matches out of 1306 query fragments, exceeding the 95–96% boundary for species assignment. TYGS clustering further assigned B38 to species cluster 1 (Figure 1), which includes *B. velezensis* and closely related species within the *Bacillus amyloliquefaciens* operational group. Based on the combined evidence from dDDH and ANI analyses, strain B38 was identified as *Bacillus velezensis* B38.

Phylogenetic tree of *Bacillus* sp. B38 and closely related type strains generated using the Type Strain Genome Server (TYGS). Heatmap columns indicate species clusters (dDDH values ≥ 70%), subspecies clusters (dDDH values ≥ 79%), G+C content, δ-statistic, genome size (bp), protein count, and the user strain. Species and subspecies clusters are distinguished by different colors; G + C content (43.76–46.62%) is represented by a blue gradient (light to dark blue, low to high values); δ-statistic by a red gradient (light to dark red, low to high phylogenetic divergence); Genome size (3,689,273–4,044,673 bp) by a black gradient (light to dark, small to large genomes); and protein count (3622–4036 proteins) by a brown gradient (light to dark, low to high protein numbers). The user strain *Bacillus* sp. B38, is indicated by a blue cross (+).

### 2.3. Antibacterial Activity Production Using PPW as Substrate

**Figure 2:**
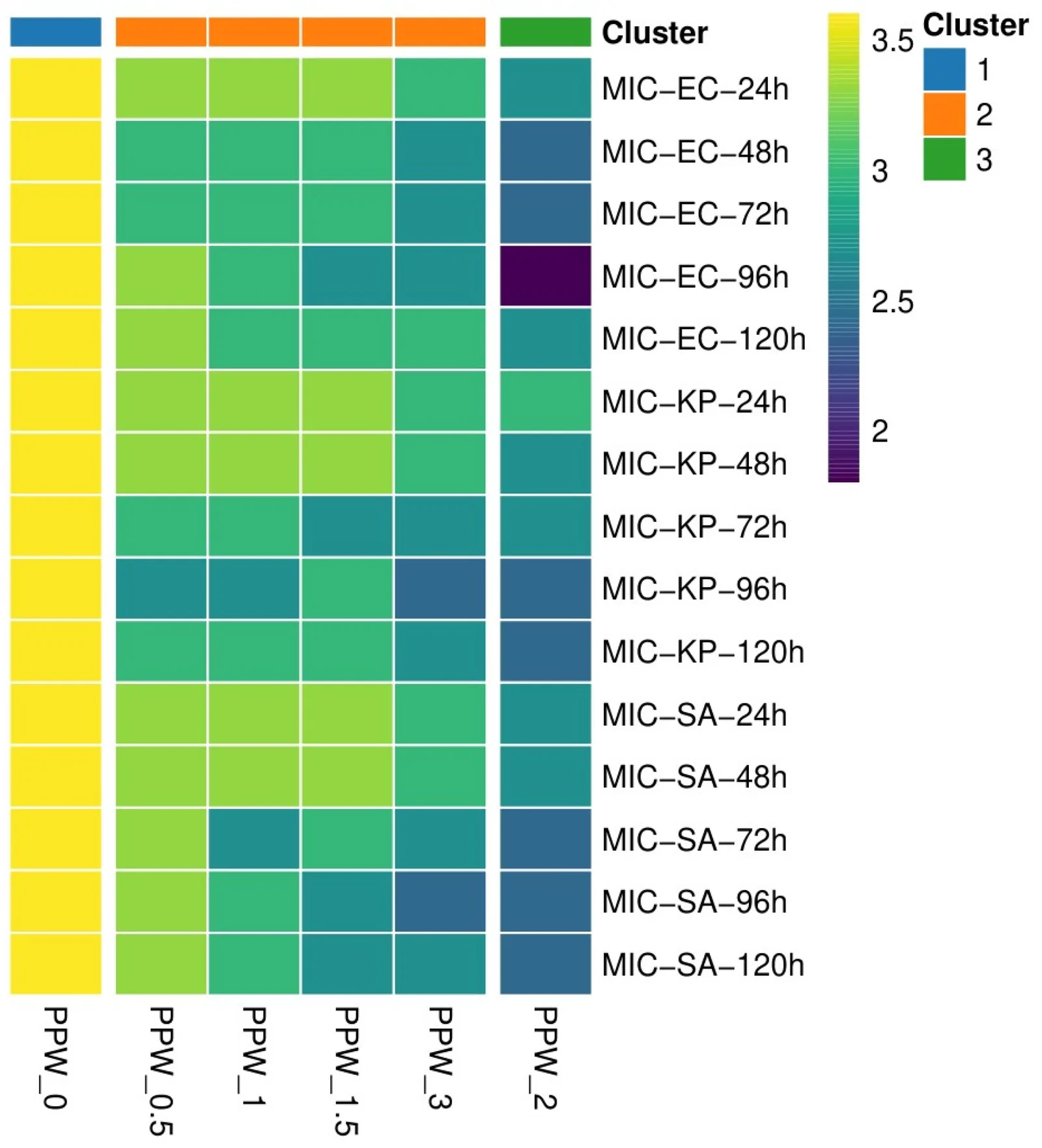
Heatmap illustrating the antibacterial activity of cell-free supernatants from *Bacillus* strain cultured with different percentages of PPW (0%, 0.5%, 1%, 1.5%, 2% and 3%) at various incubation times (24 h, 48 h, 72 h, 96 h and 120 h). The antibacterial activity was tested against *Staphylococcus aureus* 268S (SA), *Escherichia coli* 18/22 (EC) and *Klebsiella pneumoniae* 47740 (KP). Color gradients from blue to yellow indicate antibacterial activity ranging from the highest activity with the lowest MIC value (score 2) to the lowest activity with the highest MIC value (score 3.5).

The PPW was added to the fermented medium of the *Bacillus* strain at different percentages (0%, 0.5%, 1%, 1.5%, 2%, and 3%), and the antibacterial activity was monitored at different incubation times from 24 h to 120 h. The cell-free supernatant (CFS) obtained from each culture was tested against two Gram-negative bacteria (*Escherichia coli* 18/22 and *Klebsiella pneumoniae* 47740) and one Gram-positive bacterium, *Staphylococcus aureus* 268S. The addition of PPW significantly enhanced the inhibitory activity against the multi-resistant bacterial strains, whereas the control culture of *B. velezensis* B38 in LB medium without PPW supplementation did not exhibit any detectable antibacterial activity, indicating that LB medium alone was insufficient to induce the production of antibacterial metabolites. Similarly, PPW alone showed no intrinsic antibacterial activity against any of the tested bacterial strains, confirming that the observed inhibition resulted from bioactive metabolites produced during bacterial fermentation process. Furthermore, the pH of the cell-free supernatants was measured and remained close to neutrality (pH 7.0 ± 0.2). Therefore, the observed antibacterial activity could not be attributed to medium acidification. Moderate antibacterial activity was recorded in cultures supplemented with 0.5% to 1.5% PPW throughout the 5-day incubation period, with minimum inhibitory concentration (MIC) values ranging from 500 µg/mL to 2000 µg/mL. Interestingly, the addition of 2% PPW resulted in the highest antibacterial activity, displaying MIC value of 62.5 µg/mL against *E. coli* 18/22, and 250 µg/mL against *S. aureus* 268S and *K. pneumoniae* 47740 after 96 h of incubation (Figure 2). Increasing the PPW concentration to 3% did not further improve antibacterial activity, as MIC values were comparable or higher than those obtained with 2% PPW. Hierarchical clustering analysis grouped cultures supplemented with 2% PPW into a distinct cluster (cluster 3), corresponding to the highest antibacterial potency, whereas cultures containing 0.5%, 1%, 1.5%, and 3% PPW were clustered together (cluster 2), reflecting lower antibacterial activity levels (Figure 2). Consequently, 2% PPW was selected as the optimal percentage for maximal antibacterial activity production by *Bacillus velezensis* B38 strain and was used in subsequent experiments. The present study demonstrates that the inclusion of PPW in the culture medium significantly enhances the biosynthesis of bioactive compounds by *Bacillus* spp. As an agro-industrial waste product containing residual carbohydrates (28.58 g/100 g dry weight), proteins (1.552 g/100 g DW), fibers, and phenolic compounds (0.42 g/100 g DW), PPW can serve as an alternative fermentation substrate that supports microbial metabolism and promotes the biosynthesis of antibacterial metabolites by *B. velezensis* B38. Several studies have shown that agro-industrial residues such as potato peel can effectively induce metabolic synthesis in microbial fermentations. When used as substrate, PPW has been reported to enhance the production of enzymes such as amylases and cellulases [5], as well as antimicrobial lipopeptides including surfactin [26]. Optimal antibacterial activity was recorded after 96 h of incubation, corresponding to the stationary growth phase (Figure S1), indicating that the active compounds are secondary metabolites synthesized predominantly during the stationary phase. Similar fermentation kinetic studies with *Bacillus tequilensis* MSI45 have outlined that maximum antibacterial metabolite production often occurs around 96 h of incubation [27]. The complex nature of PPW may also slow substrate assimilation, extending the growth phase before the synthesis of secondary bioactive compounds [1,28].

### 2.4. Partial Purification of the Antibacterial Agents Produced by Bacillus velezensis B38 Cultured in 2% PPW

**Figure 3:**
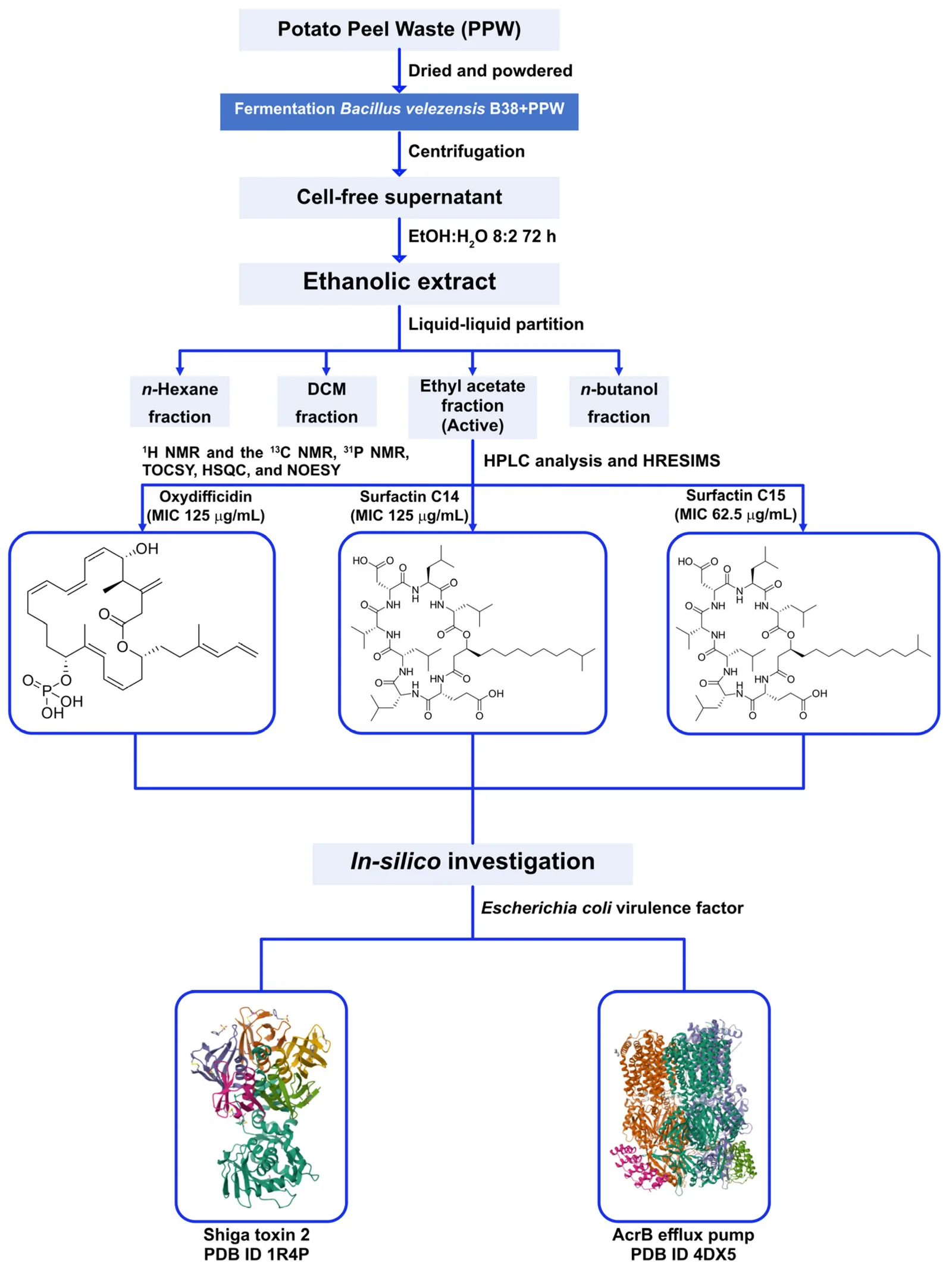
Schematic representation of Potato Peel Waste (PPW) fermentation by *Bacillus velezensis* B38, extraction steps, HPLC purification and structural characterization of the active compounds oxydifficidin, surfactin C14, surfactin C15 and molecular docking with *Escherichia coli* virulence factors: Shiga toxin and AcrB efflux pump.

The antibacterial agents present in the cell-free supernatant were initially extracted with ethanol 80%. The crude ethanolic extract exhibited significant antibacterial activity with minimum inhibitory concentration (MIC) of 62.5 µg/mL against *E. coli* 18/22. To further separate the bioactive compounds, the crude ethanolic extract was subjected to liquid–liquid partitioning using solvents of increasing polarity (Figure 3).

The ethyl acetate fraction displayed the highest antibacterial activity with MIC value of 125 µg/mL, followed by dichloromethane and butanolic fractions with moderate inhibitory effects (Table S2). The use of ethyl acetate for extracting bioactive compounds produced by *Bacillus* species is well documented, as this solvent efficiently recovers amphiphilic lipopeptides such as surfactin, fengycin, and iturin, recognized as the main agents responsible for the antimicrobial activity in *Bacillus* species [29,30]. Several studies have confirmed that the lipopeptides are more soluble in moderately polar organic solvents, particularly ethyl acetate, leading to higher recovery yields and biological activity [31]. Owing to its efficiency, ethyl acetate was selected as the optimal solvent for extracting antimicrobial compounds from *Bacillus* B38 strain and was therefore employed for subsequent analyses.

### 2.5. Antibacterial Spectrum

The ethyl acetate fraction was evaluated against a panel of Gram-positive and Gram-negative clinical bacterial isolates as well as *Candida albicans* strain (Table 1). The highest antibacterial activity was observed against *E. coli* 18/22 with a MIC and MBC values equal to 125 µg/mL, indicating a bactericidal effect at inhibitory concentration. Notable inhibitory effects were also recorded against other multidrug-resistant bacteria, including *E. coli* 12/21, *K. pneumoniae* 47740, *S. enteritidis* DMB 560 and *S. aureus* 268S with MIC values of 250 µg/mL and MBC values of 500 µg/mL. In contrast, no antifungal activity was detected against *C. albicans* ATCC 10231, with MIC value exceeding 4 mg/mL. It is noteworthy that the ethyl acetate fraction obtained from both the bacterial culture grown without 2% PPW and the PPW-supplemented medium without the bacterial inoculation did not exhibit antibacterial activity. These results confirm that the observed inhibitory effects are associated with active metabolites produced by *Bacillus velezensis* B38 strain when cultured in fermentation medium supplemented with 2% PPW and highlight the key role of PPW in enhancing the production of antibacterial compounds. The potent bactericidal activity observed against Gram-negative strains, including *E. coli*, *K. pneumoniae*, and *S. enteritidis* is especially relevant, as Gram-negative bacteria are typically more resistant to many antibacterial agents due to the presence of an outer membrane that limits compound permeability [32]. Furthermore, the significant inhibition of Gram-positive bacteria including *Staphylococcus aureus* aligns with previous findings reporting that *Bacillus*-derived lipopeptides exhibited bactericidal effect against multidrug-resistant *Staphylococcus aureus* clinical isolates [29,33]. These results suggest that the ethyl acetate fraction of *B. velezensis* B38 contains a complex mixture of bioactive molecules capable of acting on diverse bacterial targets. However, the absence of antifungal activity against *Candida albicans* suggests a lower abundance or lack of antifungal-specific compounds such as iturin, bacillomycin D or fengycins, which are typically responsible for antifungal effects in *Bacillus* species [34,35,36].

### 2.6. Time Kill Kinetics

The ethyl acetate active fraction showed a marked concentration-dependent bactericidal activity (Figure S2). Time–kill assays revealed that at 2× MIC, the extract completely killed *E. coli* 18/22 strain within 30 min. However, at MIC value, the killing effect was observed after 120 min of incubation. At half-MIC, only 87.14% reduction in viable cells was recorded after 120 min. The bactericidal effect of the ethyl acetate extract suggests the presence of membrane-active lipopeptides with strong surfactant properties, capable of interacting with the lipopolysaccharide layer of Gram-negative bacteria. Such interactions disrupt membrane integrity, increase permeability, and lead to uncontrolled leakage of intracellular constituents, ultimately resulting in cell lysis and death [37]. These mechanisms are commonly reported for *Bacillus*-derived lipopeptides, including surfactin, iturin, and fengycin [29]. Moreover, these compounds induce severe morphological alterations, membrane destabilization, and permeabilization in target pathogens, indicating that membrane disruption constitutes a primary mechanism of their antibacterial activity [38].

### 2.7. Hemolytic Activity

The ethyl acetate fraction of *Bacillus velezensis* B38 cultured in 2% PPW exhibited concentration-dependent hemolytic activity against human erythrocytes (Figure S3). At MIC (125 µg/mL), a moderate hemolytic effect was recorded (32.95% hemolysis). Increasing concentrations resulted in higher hemolytic activity, reaching approximately 46.93% at 16× MIC (2000 µg/mL). This behavior is consistent with the membrane-disrupting properties commonly reported for amphiphilic metabolites produced by *Bacillus* species, particularly lipopeptides including surfactins, iturins, and fengycins [15,39]. At higher concentration corresponding to 4000 µg/mL, a slight decrease in hemolytic activity was observed compared with 2000 µg/mL (*p* < 0.05). This reduction suggests that excessively high concentrations may promote self-assembly or micelle formation of the produced lipopeptides, thereby reducing the pool of free monomers capable of inserting into the erythrocyte membrane. This behavior aligns with previous studies showing that surfactin-like lipopeptides form micelles once their concentration exceeds the critical micelle concentration (CMC) [40,41,42,43]. Upon aggregation, the number of membrane-active monomers decreases, leading to a substantial reduction in hemolytic activity despite the increase in total concentration. The PPW extract alone exhibited lower hemolytic activity than the bacterial extract at all tested concentrations (*p* < 0.05), with a maximum hemolysis of approximately 31% at 4000 µg/mL. These results suggest that fermentation by *B. velezensis* B38 enhanced the production of membrane-active compounds compared with the non-fermented PPW extract. The hemolytic activity observed in the present study should be considered in light of the well-documented membrane-disrupting properties of surfactin-type lipopeptides. Surfactins interact with lipid bilayers leading to membrane permeabilization and consequently inducing erythrocyte lysis, a characteristic that has frequently limited their development for systemic therapeutic applications [44]. Nevertheless, despite this intrinsic hemolytic activity, surfactins continue to attract considerable interest in several non-systemic applications. In animal nutrition, surfactin-based preparations have been reported as promoting feed additives due to their antimicrobial and gut health-promoting effects [45]. In the cosmetic field, they have been explored as natural emulsifiers, preservatives and bioactive agents with anti-inflammatory properties [46]. In addition, their role in environmental applications is well established, particularly in soil bioremediation and pollutant degradation processes, where their surfactant properties facilitate the mobilization and removal of hydrophobic contaminants [47].

### 2.8. Physico-Chemical Stability of the Active Fraction

The physicochemical properties of the active fraction were evaluated, and residual antimicrobial activity was determined following exposure to thermal, enzymatic, and pH treatments (Table S3). The active fraction exhibited heat tolerance and retained the antibacterial activity after incubation at 40, 60, and 80 °C. However, a substantial reduction in antibacterial activity was recorded at 100 °C, where only 25% of the initial activity was observed. pH stability assessment showed that the active fraction maintained antibacterial activity across a broad pH range (4–12), demonstrating strong resistance to acidic and alkaline conditions. Enzymatic stability was investigated using two mammalian digestive proteases, pepsin, and chymotrypsin, applied individually and in combination. Remarkably, the active fraction retained complete antimicrobial activity under all proteolytic treatment conditions. Several antibacterial compounds produced by *Bacillus* species are recognised for their physico-chemical stability [48]. Among these molecules, cyclic lipopeptides display outstanding resistance to environmental stresses, including elevated temperatures, extreme pH values, and proteolytic degradation [49]. This stability is attributed to their specific structural features. Cyclic lipopeptides, such as surfactins, are characterized by an amphiphilic architecture consisting of a rigid cyclic peptide ring linked to a fatty acid chain, conferring high resistance to both thermal denaturation and proteolytic cleavage [50]. Their stability across a wide pH spectrum and their resistance to digestive enzymes are primarily associated with limited accessibility of protease cleavage sites.

### 2.9. Identification of the Active Compounds

High-performance liquid chromatography (HPLC)/High Resolution Electrospray Ionization Mass Spectra (HRESIMS) analysis of the ethyl acetate fraction (150 mg) revealed one active peak against *E. coli* with a retention time of 37.05 min and a MIC value of 62.5 µg/mL (Figure S4). High-resolution mass spectrometry in negative-ion mode analysis of the active peak identified three compounds: the polyketide oxydifficidin with a [M-H]^−^ peak at *m*/*z* 559.2820, and two cyclic lipopeptides, surfactins C14 and C15, composed of a cyclic heptapeptide linked to a β-hydroxy fatty acid chain containing 14 and 15 carbon atoms, respectively, and displaying [M-H]^−^ ions at *m*/*z* 1020.6593 and 1034.6740 (Figure S4). Once the three compounds were detected at the analytical level, experiments carried out on a semipreparative scale allowed the isolation of each compound (53.1 mg of oxydifficidin, 4.8 mg of surfactin C14, and 3.3 mg of surfactin C15). No commercial standards of oxydifficidin, surfactin C14, or surfactin C15 were used in this study. Instead, compound identification was achieved through HRESIMS and NMR analyses. HRESIMS data acquired in positive and negative-ion mode of surfactin C14 and C15 (Figures S5–S7), together with comparison of the ^1^H NMR and the ^13^C NMR data with those reported for oxydifficidin [51], along with analysis of its ^31^P NMR, TOCSY, HSQC, and NOESY spectra (Figures S8–S13, Table S4) confirmed the presence of these compounds.

### 2.10. Antibacterial Activity of the Purified Compounds

Following compound identification, each one was purified individually by HPLC in order to evaluate their antibacterial activity. Among the tested compounds, surfactin C15 displayed the strongest activity, with a MIC of 62.5 µg/mL, whereas oxydifficidin and surfactin C14 exhibited lower activity with a MIC 125 µg/mL (Table 2). Oxydifficidin is a polyketide antibiotic mainly produced by many species of the *Bacillus* genus, particularly by *B. velezensis* and *B*. *subtilis* [52,53,54]. Oxydifficidin produced by *B. subtilis* strains ATCC 39320 and ATCC 39374 exhibited broad-spectrum antibacterial activity against both Gram-positive and Gram-negative bacteria, including *Staphylococcus aureus*, *Streptococcus faecalis*, *Escherichia coli*, *Enterobacter cloacae*, *Klebsiella pneumoniae*, *Proteus* spp., *Morganella morganii*, and *Pseudomonas aeruginosa* [54]. Subsequent genomic and metabolomic studies also confirmed the presence of oxydifficidin biosynthetic potential in *B. subtilis* strain A1/3 [55]. Oxydifficidin-producing *B. velezensis* V4, isolated from a marine recirculation aquaculture system, showed strong inhibitory activity against fish pathogens *Aeromonas salmonicida* and *A. hydrophila*, demonstrating its relevance for aquaculture health management [56]. Additionally, supernatant extracts from *B. velezensis* B38 inhibited several clinical bacterial isolates, including *E. coli*, *K. pneumoniae*, *S. aureus*, and *S. enteritidis*, further supporting the broad antimicrobial potential of oxydifficidin-associated metabolites. On the other hand, surfactins C14 and C15 are cyclic lipopeptide biosurfactants mainly produced by *Bacillus velezensis* and closely related species such as *B. subtilis* and *B. amyloliquefaciens* [57]. They differ in the length of their β-hydroxy fatty acid chain, which influences their physicochemical and biological properties. Both compounds are synthesized by the non-ribosomal peptide synthetase encoded by the conserved srfA gene cluster [58]. Their antimicrobial activity is primarily based on disruption of cell membrane integrity by interacting with bacterial membranes through hydrophobic, dipole–dipole, and hydrogen bonding interactions [59]. They are more active against Gram-positive bacteria than Gram-negative bacteria [15]. Nevertheless, several studies have demonstrated that surfactin preparations can inhibit Gram-negative bacteria, although the reported activity varies considerably. For instance, Lilge et al. reported a 33% reduction in *Escherichia coli* growth at 100 μg/mL of a surfactin preparation [60], while Ndlovu et al. observed antibacterial effects of a surfactin-containing extract from *Bacillus amyloliquefaciens* ST34 against Gram-negative bacteria at 0.26 mg/mL, with inhibition zones ranging from 10 to 18.3 mm [61]. More recently, surfactins C11–C13 produced by *Bacillus velezensis* YA215 exhibited MIC values ranging from 7.5 to 15 μg/mL against Gram-negative pathogens [62]. The observed differences among studies likely reflect variations in surfactin structure, particularly fatty acid chain length, as well as differences in the susceptibility of the target Gram-negative strains.

### 2.11. Molecular Docking Investigations

#### 2.11.1. Docking Studies with Shiga Toxin II Subunit A

**Figure 4:**
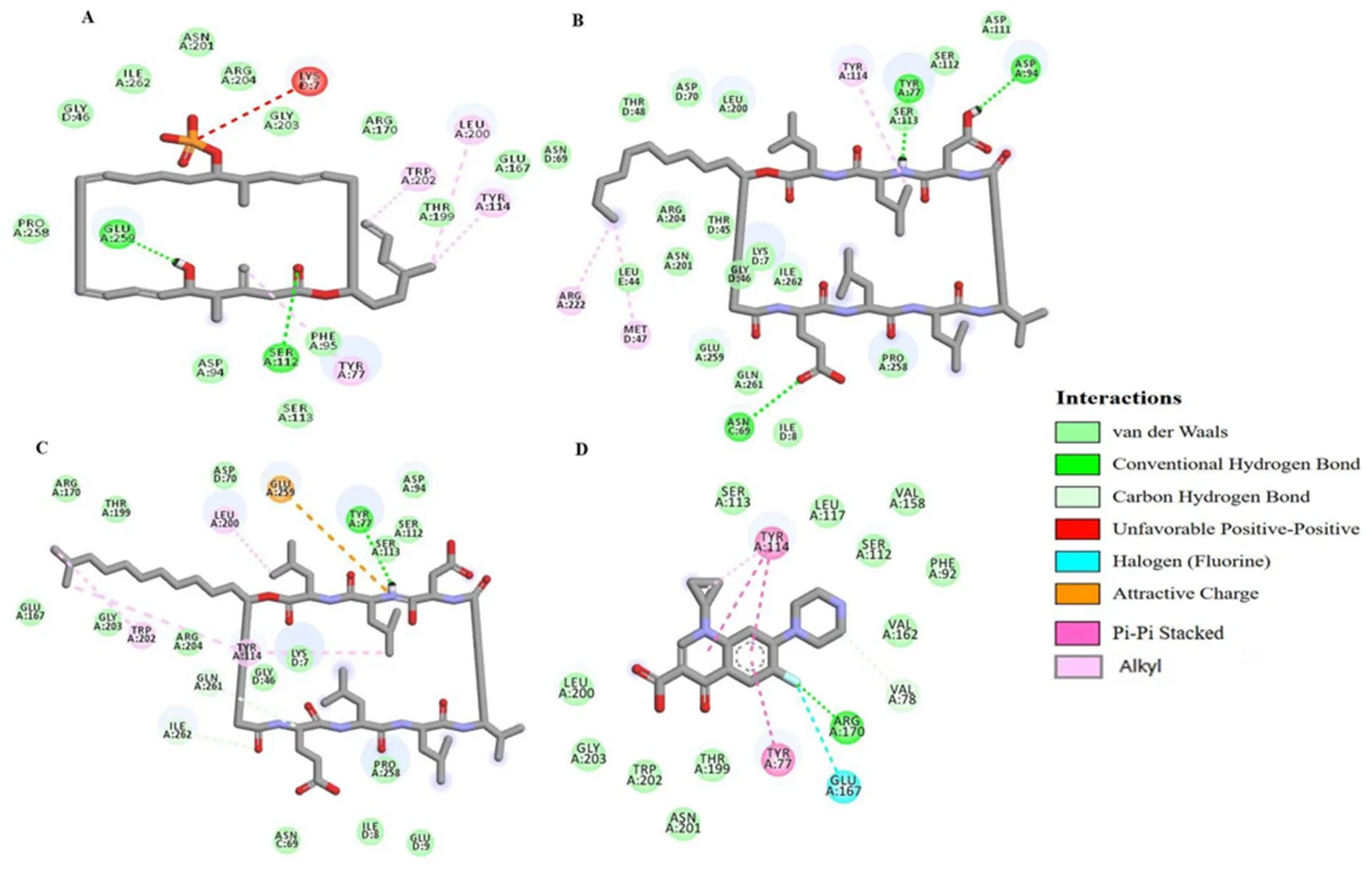
Docking analysis showing the interactions of oxydifficidin (**A**), surfactin C14 (**B**), surfactin C15 (**C**) and ciprofloxacin (**D**) with the catalytic site of Stx2 subunit A.

The isolated bioactive molecules oxydifficidin, surfactin C14, and surfactin C15 were evaluated by molecular docking analysis against Shiga toxin 2 (Stx2) subunit A, a key virulence factor of pathogenic *E. coli* strains [63] (Figure 4). Shiga toxin-producing *E. coli* (STEC) represents a heterogeneous group of pathogenic *E. coli* strains producing Shiga toxins (Stxs). These toxins inhibit protein synthesis in host cells, leading to cell damage and playing a pivotal role in the development of severe clinical outcomes. STEC infections are associated with a broad-spectrum of diseases, including diarrhea, hemorrhagic colitis (HC), and hemolytic uremic syndrome (HUS) in humans, as well as gastrointestinal illness, edema disease in swine, wildlife infections, calf mortality, diarrhea, and dysentery in calves [64,65]. Among the two main toxin variants, Stx2 is recognized as more virulent and more frequently associated with severe complications compared to Stx1 [66]. Given its central role in host–cell damage and disease complications, Stx2 constitutes a critical molecular target for the development of novel therapeutic strategies. The identified molecules were subjected to *in silico* studies to investigate potential interaction with Stx2 virulence factor (Table 3). Among the tested compounds, oxydifficidin exhibited the most favorable predicted binding affinity toward Shiga toxin Stx2 subunit A, with a notable binding energy of −9.3 kcal/mol, followed by surfactin C15 with a predicted binding affinity comparable to that of ciprofloxacin (−8.5 kcal/mol). However, surfactin C14 showed the lowest affinity with a binding energy of −8.0 kcal/mol. The docking analysis suggested that oxydifficidin and surfactin C15 may interact with key residues located within the catalytic pocket of Shiga toxin subunit A, including Tyr77, Asp94, Ser112, Tyr114, Glu167, Arg170, Thr199, and Gly203 (Figure 4A). The predicted interactions involving Glu167 and Asp94 indicate that these molecules could potentially cover a wide region of the catalytic pocket from the bottom of the cavity (Glu167) to the gate area (Asp94) [67]. In contrast, surfactin C14 interacted only with part of the catalytic pocket, including Tyr77, Asp94, Ser112, and Tyr114 (Figure 4B). As expected from their structural similarity, surfactin C14 and C15 were predicted to interact with several common residues through their conserved peptide ring. However, surfactin C15 established additional predicted contacts involving Glu167, Arg170, Thr199, and Gly203 through its longer acyl chain (Figure 4C). These observations suggest that differences in acyl chain length may influence the affinity toward Shiga toxin subunit A. Moreover, ciprofloxacin, used as a conventional antibiotic, interacted with a similar set of residues as surfactin C15, except Asp94 and Glu167 (Figure 4D). The docking results suggest a potential interaction of oxydifficidin and surfactin C15 with the catalytic pocket of Shiga toxin II subunit A and indicate a theoretical affinity for this target.

#### 2.11.2. Docking Studies with AcrB of AcrAB-TolC Tripartite Efflux Pump

**Figure 5:**
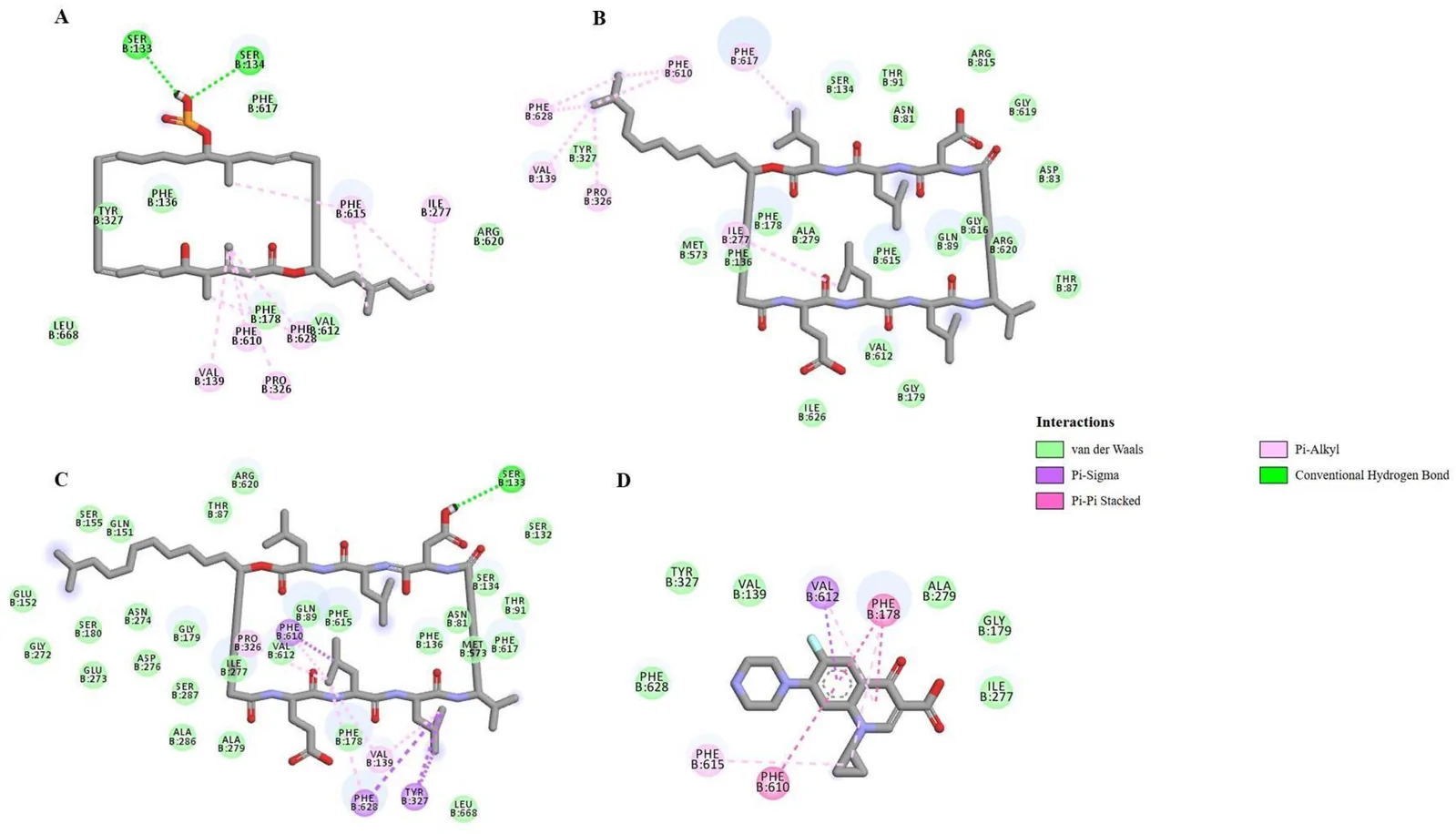
Docking analysis showing the interactions of oxydifficidin (**A**), surfactin C14 (**B**), surfactin C15 (**C**) and ciprofloxacin (**D**) with the AcrB binding site.

The widespread antibiotic resistance observed in pathogenic *E. coli* strains is largely attributed to the AcrAB-TolC tripartite efflux pump. Within this system, the inner membrane transporter AcrB plays a pivotal role in antibiotic resistance by recognizing and exporting a wide range of antibiotics through the AcrAB–TolC efflux system. Because interactions with AcrB can influence the intracellular accumulation of antibiotics, the binding of the identified bioactive compounds, namely oxydifficidin, surfactin C14, and surfactin C15 to AcrB was assessed through molecular docking analysis (Figure 5, Table 4). Oxydifficidin exhibited the most favorable predicted binding affinity, with ΔG value of −11.4 kcal/mol. Likewise, surfactin C14 and C15 showed predicted binding affinities of −9.6 and −10.3 kcal/mol, respectively. In contrast, ciprofloxacin, used as the conventional antibiotic, displayed the lowest predicted binding energy of −8.6 kcal/mol.

The docking results suggest that oxydifficidin and both surfactin homologues may interact with several residues within the AcrB binding pocket, including those residues located in the cave region rich in hydrophobic residues Phe136, Val139, Tyr327, Val571 and Phe628 and the groove region comprising Phe178, Ile277, Val612, and Phe615 [68]. Importantly, the identified compounds were predicted to establish interactions with phenylalanine residues containing the hydrophobic trap (HP-trap: Phe136, Phe178, Phe610, Phe615, and Phe628) (Figure 5), a region that has been proposed as an important binding site for AcrB inhibitors [68,69,70]. The relatively favorable predicted binding energies of surfactins and oxydifficidin compared with ciprofloxacin may be attributed to their hydrophobic character. Their structural features, comprising both aliphatic chains and hydrophobic peptide or polyketide scaffolds, could promote hydrophobic/alkyl interactions, as well as hydrogen interactions within the AcrB hydrophobic trap and the deep binding pocket of the tight monomer (DP_T_), a prominent recognition site of AcrB. These interactions likely stabilize these compounds within the efflux pump’s binding pocket. Similar interactions have been previously reported for the active compounds affinine, reserpine, and verapamil which interact with the AcrB binding pocket to modulate efflux activity, thereby enhancing intracellular antibiotic accumulation and reducing MIC values when used in combination therapies [71]. Further studies, including combination assays with conventional antibiotics, will be conducted to validate this hypothesis. In this context, previous studies have reported that the antibiotic adjuvant domperidone, an AcrB inhibitor, can be used to treat potentially fatal infections caused by MDR levofloxacin and ciprofloxacin resistant *E. coli* pathogens [72]. The docking results further indicate that ciprofloxacin, a smaller and more polar molecule, may establish fewer hydrophobic interactions within AcrB binding pocket than oxydifficidin and surfactin homologues, resulting in a less favorable predicted binding energy (Figure 5D). This is consistent with prior findings showing that hydrophilic carbapenems interact weakly with the hydrophobic DP_T_ of AcrB [68], making them more readily expelled by the pump.

## 3. Material and Methods

### 3.1. Bacterial Strains

*Bacillus* strain B38 was used as the antibacterial-producing strain. This strain was isolated from soil and was identified based on the whole genome sequencing, performed in collaboration with the Sainsbury Laboratory (Norwich, UK) within the framework of the Get Genome—Genomics for All programs [73]. The whole genome sequence of the strain B38 has been deposited in the DDBJ/ENA/GenBank database under the accession number JBAKJE000000000. Pathogenic Gram-negative bacteria including *Escherichia coli* 18/22 (Table S5), *E. coli* 19/21 and *Klebsiella pneumoniae* 47740, as well as a Gram-positive strain *Staphylococcus aureus* 268S were used as indicator strains to assess the antibacterial activity spectrum. These pathogenic strains were kindly provided by the Laboratory of Clinical Biochemistry, Mohamed Taher Maamouri Hospital, Nabeul, Tunisia. *Salmonella enteritidis* DMB 560 and *Candida albicans* ATCC 10231 were obtained from the microbial culture collection of the Laboratory of Bioactive compounds, Biotechnology Center of Borj Cedria.

### 3.2. Whole-Genome-Based Taxonomic Analysis

Whole-genome sequence data were analyzed using the Type (Strain) Genome Server (TYGS; https://tygs.dsmz.de, accessed on 22 December 2025) for taxonomic assignment. Closely related type strains were identified through MASH-based whole-genome comparisons and 16S rRNA gene sequence analysis, with 16S rRNA gene sequences extracted using RNAmmer. Intergenomic distances were calculated using the Genome BLAST Distance Phylogeny (GBDP) approach with distance formula d5. Digital DNA–DNA hybridization (dDDH) values were determined with the Genome-to-Genome Distance Calculator (GGDC 4.0). Phylogenomic relationships were inferred from a balanced minimum evolution tree constructed with FASTME, supported by 100 bootstrap replicates, midpoint-rooted, and visualized with PhyD3. Species and subspecies were delineated using dDDH thresholds of 70% and 79%, respectively. Average Nucleotide Identity (ANI) was calculated via the Proksee server (https://proksee.ca, accessed on 22 December 2025) to further confirm species assignment.

### 3.3. Potato Peel Waste (PPW) Preparation and Chemical Composition

Potato tubers (*Solanum tuberosum* L., cv. Spunta) were purchased from the local market, and individually washed with tap water. The tubers were then peeled, and the resulting peels were lyophilized, ground, and stored at −20 °C until further use. For moisture content determination, PPs were dehydrated in an oven at 105 °C until constant weight. Total sugars were quantified using the colorimetric method [74]. Protein content was estimated by the kjeldahl method by multiplying the measured nitrogen (N) content by 6.25. Ash content was determined by calcination at 550 °C. Total phenolic and flavonoid contents were quantified as previously described [75].

### 3.4. Culture Conditions

*Bacillus* strain B38 was cultured in LB broth (Tryptone 10 g/L, Yeast extract 5 g/L and NaCl 10 g/L) supplemented with different concentrations of PPW powder (0%, 0.5%, 1%, 2%, and 3%). Cultures were incubated for 5 days in a shaking incubator (Daihan Labtech Co., Ltd., Namyangju, Republic of Korea) at 30 °C and 150 rpm. At 24 h intervals, 1 mL aliquots were collected from each treatment, and cell free supernatants were obtained by centrifugation (Sorvall ST16R centrifuge, Thermo Electron LED GmbH, Osterode am Harz, Germany) at 12,000 rpm for 15 min at 4 °C. The supernatants were stored at −20 °C until further analysis. Bacterial growth was monitored throughout the 5-day incubation period by determining viable cell counts at 24 h intervals. Colony forming units (CFU/mL) were quantified by serial dilution and plate counting. The following control treatments were included: (i) *Bacillus velezensis* B38 grown in LB broth without PPW supplementation, (ii) uninoculated LB-broth supplemented with PPW, and (iii) uninoculated LB broth without PPW.

### 3.5. Extraction and Identification of the Active Compounds

The active compounds were purified from the cell-free supernatant following the protocol described in Figure 3. Cell free supernatant was lyophilized and then extracted with 80% ethanol. The crude extract was concentrated under reduced pressure using a rotary evaporator and subsequently fractionated by sequential liquid–liquid partitioning with solvents of increasing polarity: *n*-hexane, dichloromethane, ethyl acetate, and *n*-butanol. Each organic fraction was evaporated under reduced pressure at 45 °C, re-dissolved in 80% aqueous ethanol, and evaluated for antibacterial activity. Among the solvent fractions obtained following sequential liquid–liquid partitioning of the crude ethanolic extract, the ethyl acetate fraction exhibiting the strongest antibacterial activity was selected for further purification. Hence, the ethyl acetate fraction was further analyzed by High-Performance Liquid Chromatography (HPLC) using a Luna^®^ Omega reverse-phase C18 chromatographic column (250 mm × 10 mm, 5 μm). The mobile phase consisted of Milli-Q water containing 0.1% formic acid (A) and acetonitrile (B). Elution was performed using the following gradient program: 0–5 min, 0–20% B; 5–10 min, 20–30% B; 10–15 min, 30–50% B; 15–25 min, 50–70% B; 25–35 min, 70–100% B; 35–45 min, 100–50% B; and 45–50 min, 50–20% B. The flow rate was maintained at 1.5 mL/min, and detection was carried out at 254 nm using a UV detector. High-resolution mass spectrometry (HR-MS) analysis was performed to determine the molecular masses and composition of compounds collected from the active HPLC fractions. LC/HR-MS data were acquired using an LTQ Orbitrap Discovery mass spectrometer coupled to an Accela HPLC system (Thermo Fisher Scientific, Waltham, MA, USA) in negative ion full scan mode over a *m*/*z* range of 100–2000 under electrospray ionization (ESI) conditions. Structural elucidation was further confirmed by nuclear magnetic resonance (NMR) spectroscopy using a Bruker Avance 500 spectrometer (Bruker BioSpin GmbH & Co. KG, Rheinstetten, Germany). One-dimensional ^1^H (500 MHz), ^13^C (125 MHz), and ^31^P (202 MHz) NMR spectra were performed. Two-dimensional NMR analyses, including TOCSY, HSQC, and NOESY were carried out using standard pulse sequences. NMR spectral data were processed using MestReNova (Mestrelab Research S.L., Santiago de Compostela, A Coruña, Spain) software (version 14.1). Chemical shifts (δ) were reported in parts per million (ppm) and referenced to CD_3_OD (δ = 3.31 ppm for ^1^H, δ = 49.0 ppm for ^13^C).

### 3.6. Physico-Chemical Stability of the Active Extract

Thermal stability was assessed by exposing the active extract to temperatures of 40, 60, 80, and 100 °C for 2 h in a water bath, after which residual antibacterial activity was tested against *E. coli* 18/22 [22]. For pH stability, the extract was adjusted to pH 4–12 using 10 mM HCl or NaOH, incubated at room temperature for 2 h, then readjusted to pH 6 before activity assessment [76]. Proteolytic stability was evaluated through sequential digestion with pepsin (0.025%, pH 2.0) and chymotrypsin (100 mM Tris-HCl, pH 8.0), each for 2 h at 37 °C [77,78]. Enzymatic reactions were achieved by heating at 90 °C for 5 min, followed by centrifugation at 10,000 rpm for 10 min, and the recovered supernatant was tested for residual activity. Each enzyme was also assessed independently.

### 3.7. Determination of the Minimal Inhibitory Concentration (MIC) and the Minimal Bactericidal Concentration (MBC)

The antibacterial activity was evaluated using the broth microdilution method according to the Clinical and Laboratory Standards Institute (CLSI) guidelines [79]. Twofold serial dilutions of each tested sample were prepared in sterile 96-well microplate containing Mueller-Hinton (MH) broth (Bio Basic, Markham, ON, Canada). Bacterial inocula were prepared from fresh cultures and adjusted to 0.5 McFarland standard (approximately 10^8^ CFU/mL), then diluted in MH broth to obtain a final inoculum concentration of approximately 5 × 10^5^ CFU/mL in each well. Following overnight incubation at 37 °C, the MIC was determined as the lowest concentration of the tested sample that inhibited visible bacterial growth. The MBC was also determined by culturing the content of wells with no apparent growth onto MHA plates and subsequent incubation at 37 °C for further 24 h. The lowest concentration that inhibited colony formation on agar plates was considered as MBC of the tested sample.

### 3.8. Killing Kinetics

Time-kill assay was performed against *Escherichia coli* 18/22 at different concentrations: ½× MIC, MIC, and 2× MIC corresponding to 62.5, 125 and 250 µg/mL, respectively, as previously described [80]. *E. coli* 18/22 cells were adjusted to an initial inoculum of 10^6^ CFU/mL in LB broth and incubated with the active extract concentrations at 37 °C. At predetermined time intervals, 10 µL aliquots were collected, serially diluted, and plated onto LB agar. Plates were incubated at 37 °C for 24 h and bacterial colonies were counted. Viable cells (CFU/mL) were calculated and compared with those of the untreated control culture.

### 3.9. Hemolytic Assay

The hemolytic activity was evaluated using human erythrocytes, provided by the Laboratory of Clinical Biochemistry, Mohamed Taher Maamouri Hospital, Nabeul, Tunisia [81]. Briefly, aliquots of human erythrocyte suspension (10^7^ cells/mL in 0.9% NaCl) were incubated with various concentrations of the extracts. Following incubation at 37 °C for 30 min, samples were centrifuged at 3500 rpm for 15 min, and the supernatant was measured at 450 nm. Complete hemolysis, obtained by suspending erythrocytes in distilled water, was used as the positive control. All experiments were performed in triplicate, and the percentage of hemolysis was calculated.

### 3.10. Molecular Docking Analysis

The crystal structures of the target proteins, Shiga toxin 2 (Stx2) subunit A (PDB ID: 1R4P) and AcrB from the AcrAB–TolC efflux pump (PDB ID: 4DX5) were retrieved from the Protein Data Bank [68,82]. The three-dimensional structures of the active compounds oxydifficidin, surfactin C14, and surfactin C15 as well as the conventional antibiotic ciprofloxacin were obtained from the PubChem database (Table S6). Ligand structures were optimized using ACD/3D Viewer (Freeware, version 2017.2.1; ACD/Labs, Advanced Chemistry Development, Inc., Toronto, ON, Canada) and converted to the PDBQT format using AutoDock Tools v1.5.6. Prior to docking, heteroatoms and crystallographic water molecules were removed from the protein structures. Polar hydrogens were added and Kollman charges were assigned using AutoDock Tools (ADT), and the prepared protein structures were saved in PDBQT format. Molecular docking simulations were carried out using AutoDock 1.5.6rc3 vina tools. The empirical Vina scoring function was employed to estimate ligand–protein binding affinities, expressed as predicted binding free energies (kcal/mol). Docking calculations were carried out using an exhaustiveness value of 8, and up to 9 binding poses were generated for each ligand. The binding pose exhibiting the lowest predicted binding energy was selected for subsequent interaction analysis and visualization. The docking search space was defined around the active site of each target protein using the grid box parameters. The docking grid boxes and binding sites were defined based on previously reported active sites for Stx2 subunit A [65] and AcrB of the AcrAB–TolC efflux pump [82] (Table S7). Docking calculations were performed to predict the most favorable binding orientations and conformations of the ligands within the target protein active sites. The docking protocol was validated by redocking the native co-crystallized ligands (MMA-beta Ala peptide and Minocycline) into the active sites of the respective reference protein structures of Shiga toxin 2 and AcrB of the AcrAB–TolC efflux pump. The superposition of the crystallographic and predicted poses yielded an RMSD value below 2.0 Å, confirming the reliability of the docking procedure and reinforcing the validity of our molecular docking results and the expected binding affinities. The optimal docking poses were selected based on the lowest binding free energy (ΔG) values. Protein–ligand interactions were visualized and analyzed using Discovery Studio Visualizer v17.2.0.16349, enabling detailed characterization of hydrogen bonding, hydrophobic interactions, and binding pocket occupancy.

### 3.11. Statistical Analysis

All experiments were conducted in triplicate, and data were expressed as mean values ± standard deviations (SD). Statistical analyses were carried out using RStudio (version 4.5.2). Significant differences among treatments were evaluated using one-way analysis of variance (ANOVA), followed by Tukey’s Honest Significant Difference (HSD) post hoc test for multiple comparisons. Statistical significance was set at *p* < 0.05. Data processing and graphical visualization were performed using ggplot2 and multcompView packages.

## 4. Conclusions

In conclusion, this study represents an interesting prospect utilizing PPW, as an effective low-cost fermentation substrate for enhancing the capacity of *Bacillus velezensis* B38 to produce antibacterial metabolites including surfactins and oxydifficidin active against pathogenic *E. coli*. The obtained results highlight the potential of PPW-*B. velezensis* B38 bioprocess for the generation of value-added antimicrobial metabolites from agro-industrial residues. In addition, molecular docking analysis provided further insights into the possible mechanisms underlying the antivirulence activities of the identified metabolites. Further studies are required to optimize large-scale production conditions, develop suitable formulations for potential application as animal feed additives, and evaluate the safety, stability, and in vivo efficacy of these bioactive compounds under practical application conditions.

## Figures and Tables

**Table 1:** Antibacterial spectrum of the ethyl acetate fraction from *B. velezensis* B38 cultured in 2% PPW.

Strains	Antimicrobial Activity	
	MIC (µg/mL)	MBC (µg/mL)
*Escherichia coli* 18/22	125	125
*Escherichia coli* 12/21	250	500
*Staphylococcus aureus* 268S	250	500
*Klebsiella pneumonia* 47740	250	500
*Salmonella enteritidis* DMB 560	250	500
*Candida albicans* ATCC 10231	>4000	>4000

**Table 2:** Identification of antibacterial compounds produced by *B. velezensis* B38 in the fermentation medium supplemented with 2% PPW by (-) HR-ESI-MS analysis, including molecular formula, observed and calculated *m*/*z* values, structural assignments and minimal inhibitory concentration (MICs) against *E. coli* 18/22.

Identified Compounds	Ion	Formula	*m*/*z* Observed	*m*/*z* Calculated	Structure	MIC (µg/mL)
Oxydifficidin	[M-H]^−^	C_31_H_44_O_7_P	559.2820	559.2819	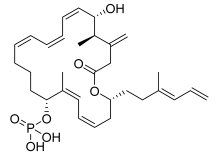	125
Surfactin C14	[M-H]^−^	C_52_H_90_N_7_O_13_	1020.6593	1020.66	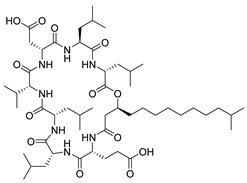	125
Surfactin C15	[M-H]^−^	C_53_H_92_N_7_O_13_	1034.6740	1034.68	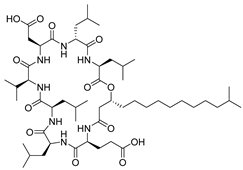	62.5

MIC: Minimum Inhibitory Concentration values expressed in µg/mL against the multidrug-resistant *E. coli* 18/22 strain.

**Table 3:** Molecular docking results showing the binding energies (kcal/mol) of Stx2 subunit A docked with oxydifficidin, surfactin C14 and surfactin C15. Ciprofloxacin was used as the conventional antibiotic.

Receptor	Ligands	Binding Energy (kcal/mol)	Active Site Amino Acids	Interaction Type
Stx2	Oxydifficidin	−9.3	A/SER.112, A/GLU.259	Hydrogen
			A/TYR.77, A/TYR.114, A/TRP.202, A/LEU.200	Alkyl
			D/LYS.7	Unfavorable positive
			A/ASP.94, A/PHE.95, A/SER.113, A/GLU.167, A/ARG.170, A/THR.199, A/ASN.201, A/GLY.203, A/ARG.204, A/PRO.258, A/ILE.262, D/GLY.46	Van der Waals
	Surfactin C14	−8.0	A/TYR.77, A/ASP.94, C/ASN.69	Hydrogen
			A/ASP.94, A/SER.112, A/SER.113, A/LEU.200, A/ASN.201, A/ARG.204, A/ARG.222, A/PRO.258, A/GLU.259, A/GLN.261, A/ILE.262, D/LYS.7, D/ILE.8, D/THR.45, D/GLY.46 D/THR.48, D/ASN.69, D/ASP.70, E/LEU.44	Van der Waals
			A/TYR.114, A/MET.47, A/ARG.222	Alkyl
	Surfactin C15	−8.5	A/TYR.77	Hydrogen
			A/ASP.94, A/SER.112, A/SER.113, A/GLU.167, A/ARG.170, A/THR.199, A/ASN.201, A/GLY.203, A/ARG.204, A/PRO.258, A/GLN.261, C/ASN.69, D/LYS.7, D/ILE.8, D/GLY.46, D/ASP.70	Van der Waals
			A/TYR.114, A/LEU.200, A/TRP.202	Alkyl
			A/ILE.262	Carbon-hydrogen bond
			A/GLU.259	Attractive charge
	Ciprofloxacin	−8.5	A/ARG.170	Hydrogen
			A/TYR.77, A/TYR.114	Pi-Pi stacked
			A/GLN.261	Halogen
			A/VAL.78, A/PHE.92, A/SER.101, A/SER.113, A/LEU.117, A/VAL.158, A/VAL.162, A/THR.199, A/ASN.201, A/TRP.202, A/GLY.203	Van der Waals

**Table 4:** Molecular docking results showing the binding energies (kcal/mol) of acrB of the AcrAB-TolC efflux pump docked with oxydifficidin, surfactin C14 and surfactin C15. Ciprofloxacin was used as the conventional antibiotic.

Receptor	Ligands	Binding Energy (kcal/mol)	Active Site Amino Acids	Interaction Type
AcrAB-TolC	oxydifficidin	−11.4	B/SER.133, B/SER.134	Hydrogen
			B/PHE.617, B/PHE.136, B/PHE.178, B/VAL.612, B/ARG.620, B/LEU.668, B/TYR.327	Van Der Waals
			B/ILE.277, B/PHE.615, B/PHE.610, B/PHE.628, B/PRO.326, B/VAL.139	Alkyl
	Surfactin C14	−9.6	B/ASN.81, B/THR.87, B/GLN.89, B/THR.91, B/SER.134, B/PHE.136, B/PHE.178, B/ALA.279, B/MET.573, B/VAL.612, B/PHE.615, B/GLY.616, B/PHE.617, B/ARG.620	Van Der Waals
			B/VAL.139, B/ILE.277, B/PRO.326, B/THR.327, B/PHE.610, B/PHE.628	Alkyl
	Surfactin C15	−10.6	B/SER.134	Hydrogen
			B/ASN.81, B/THR.87, B/GLN.89, B/THR.91, B/SER.133, B/PHE.136, B/GLN.151, B/GLN.152, B/SER.155, B/PHE.178, B/GLY.272, B/GLU.273, B/ASN.274, B/ASP.276, B/ALA.279, B/ALA.286, B/SER.287, B/MET.573, B/VAL.612, B/PHE.615, B/PHE.617, B/ARG.620	Van Der Waals
			B/VAL.139, B/PRO.326	Alkyl
			B/PHE.610, B/TYR.327, B/PHE.628	Pi-sigma
	ciprofloxacin	−8.6	B/VAL139, GLY.179, B/ILE.277, ALA.279, B/TYR.327, B/PHE.628	Van Der Waals
			B/VAL.612	Pi-Sigma
			B/PHE.615	Pi-Alkyl
			B/PHE.178, B/610	Pi-Pi stacked

## Data Availability

The original contributions presented in this study are included in the article/Supplementary Materials. Further inquiries can be directed to the corresponding authors.

## Supplementary Materials

The following supporting information can be downloaded at https://www.mdpi.com/article/10.3390/molecules31162747/s1, Figure S1: Growth kinetics of *Bacillus velezensis* B38 and corresponding antibacterial activity against *Escherichia coli* 18/22 in medium supplemented with 2% potato peel waste (PPW); Figure S2: Time-kill kinetics of ethyl acetate extract obtained from *Bacillus velezensis* B38 cultivated with 2% PPW against *Escherichia coli* 18/22; Figure S3: The hemolytic activity against human erythrocytes; Figure S4: HPLC profile of the active fraction at 254 nm; Figure S5: (-) HR-ESI mass spectrum of the active peak; Figure S6: (+)-HRESIMS of surfactin C14; Figure S7: (+)-HRESIMS of surfactin C15; Figure S8: 1H NMR spectrum (500 MHz, CD_3_OD) of oxydifficidin; Figure S9: 13C NMR spectrum (125 MHz, CD_3_OD) of oxydifficidin; Figure S10: 31P NMR spectrum of oxydifficidin; Figure S11: HSQC spectrum of oxydifficidin; Figure S12: TOCSY spectrum of oxydifficidin; Figure S13: NOESY spectrum of oxydifficidin; Table S1: Composition of potato peel (PPW) powder; Table S2: Partial purification of antibacterial agents from *Bacillus velezensis* B38 cultured in 2% PPW; Table S3: Physico-chemical characterization of the ethyl acetate active fraction of *Bacillus velezensis* B38 cultured in 2% PPW; Table S4: NMR data of oxydifficidin, 500 MHz, CD_3_OD; Table S5: Resistance profile of *Escherichia coli* 18/22 strain; Table S6: Oxydifficidin, surfactin C14, surfactin C15, and ciprofloxacin characteristics and structures obtained from PubChem; Table S7: Grid box parameters of target ligands: the stx2 subunit A and AcrB of the AcrAB-TolC efflux pumps.

## Abbreviations

^13^C NMR	Nuclear Magnetic Resonance of Carbon 13
^1^H NMR	Nuclear Magnetic Resonance of Hydrogen 1
ABTS	2,2′-Azino-bis (3-ethylbenzothiazoline-6-sulfonic acid)
AcrA	Acriflavine resistance protein A
AcrB	Acriflavine resistance protein B
ADT	AutoDock Tools
ANI	Average Nucleotide Identity
CFU	Colony Forming Units
CMC	Critical Micelle Concentration
COSY	Correlation Spectroscopy
DPPH	2,2-Diphenyl-1-picrylhydrazyl
DW	Dry weight
GAE	Gallic Acid Equivalents
HMBC	Heteronuclear Multiple Bond Correlation
HSQC	Heteronuclear Single Quantum Coherence
LB broth	Luria–Bertani broth
LC/HR-ESI-MS	Liquid Chromatography/High-Resolution Electrospray Ionization Mass Spectrometry
MBC	Minimal Bactericidal Concentration
MH broth	Mueller-Hinton broth
MIC	Minimal Inhibitory Concentration
NOESY	Nuclear Overhauser Effect Spectroscopy
NRPSs	Non-Ribosomal Peptide Synthetases
PDB IDs	Protein Data Bank Identifiers
PDBQT	Protein Data Bank, Partial Charge (Q), and Atom Type (T) format
PKSs	polyketide synthases
PPW	Potato peel waste
RMSD	Root Mean Square Deviation
STEC	Shiga toxin-producing *Escherichia coli*
TolC	Tolerance protein C
TPC	Total phenolic content
TYGS	Type Strain Genome Server
UPEC	Uropathogenic *Escherichia coli*
