# Supplementary material for: Potato (Solanum tuberosum L., cv. Spunta) Peels Promote the Production of Broad Spectrum Antibacterial Chemicals by Bacillus velezensis B38
Source: Molecules. 2026 Aug 7;31(16):2747. doi: 10.3390/molecules31162747 (PMC13515681; doi:10.3390/molecules31162747)
Supplement: Supplementary file 1 [file molecules-31-02747-s001.zip › molecules-4342988-supplementary.pdf]

Supplementary Material

Potato (*Solanum tuberosum* L., cv. Spunta) Peels Promote the Production of Broad Spectrum Antibacterial Chemicals by *Bacillus velezensis* B38

Malek Gharbi [1,2], Dorra Gharbi [2], Ines Karkouch [2], Abel M. Forero [3], Jaime Rodríguez [3], Manel Chaouachi [2], Takwa Marzouk [2], Asma Bachali [4], Carlos Jiménez [3] and Olfa Tabbene [2,*]

1. Faculty of Sciences of Tunis, University of Tunis El Manar, Tunis 2092, Tunisia; malek.gharbi@cbbc.rnrt.tn
2. Laboratoire des Substances Bioactives, Centre de Biotechnologie de Borj Cedria, BP 901, Hammam Lif 2050, Tunisia; dorra.gharbi@cbbc.rnrt.tn (D.G.); ines.karkouch@cbbc.rnrt.tn (I.K.); manel.chaouachi@cbbc.rnrt.tn (M.C.); takwa.marzouk@cbbc.rnrt.tn (T.M.)
3. Centro Interdisciplinar de Química e Bioloxía (CICA) and Departamento de Química, Facultade de Ciencias, Universidade da Coruña, 15071 Coruña, Spain; mateo.forerot@udc.es (A.M.F.); jaime.rodriguez@udc.es (J.R.); carlos.jimenez@udc.es (C.J.)
4. Laboratory of Clinical Biochemistry, Mohamed Taher Maamouri Hospital, Nabeul 8000, Tunisia; asma.bachali@fmt.utm.tn

* Correspondence: olfa.tabbene@cbbc.rnrt.tn; Tel.: +216-79-325-511; Fax: +216-79-325-638

Table of Contents

Figure S1. Growth kinetics of *Bacillus velezensis* B38 and corresponding antibacterial activity against *Escherichia coli* 18/22 in medium supplemented with 2% potato peel waste (PPW).. 2
Figure S2. Time-kill kinetics of ethyl acetate extract obtained from *Bacillus velezensis* B38 cultivated with 2% PPW against *Escherichia coli* 18/22. 3
Figure S3. The hemolytic activity against human erythrocytes. 4
Figure S4. HPLC profile of the active fraction at 254 nm 4
Figure S5. (-) HR-ESI mass spectrum of the active peak 5
Figure S6. (+)-HRESIMS of surfactin C14 5
Figure S7. (+)-HRESIMS of surfactin C15 6
Figure S8. ^{1}H NMR spectrum (500 MHz, CD_3OD) of oxydifficidin 7
Figure S9. ^{13}C NMR spectrum (125 MHz, CD_3OD) of oxydifficidin 8
Figure S10. ^{31}P NMR spectrum of oxydifficidin 9
Figure S11. HSQC spectrum of oxydifficidin 10
Figure S12. TOCSY spectrum of oxydifficidin 11
Figure S13. NOESY spectrum of oxydifficidin 12
Table S1. Composition of potato peel (PPW) powder 13
Table S2. Partial purification of antibacterial agents from *Bacillus velezensis* B38 cultured in 2% PPW 13
Table S3. Physico-chemical characterization of the ethyl acetate active fraction of *Bacillus velezensis* B38 cultured in 2% PPW 13
Table S4. NMR data of oxydifficidin, 500 MHz, CD_3OD 14
Table S5. Resistance profile of *Escherichia coli* 18/22 strain 15
Table S6. Oxydifficidin, surfactin C14, surfactin C15, and ciprofloxacin characteristics and structures obtained from PubChem 15
Table S7. Grid box parameters of target ligands: the stx2 subunit A and AcrB of the AcrAB-TolC efflux pumps 15

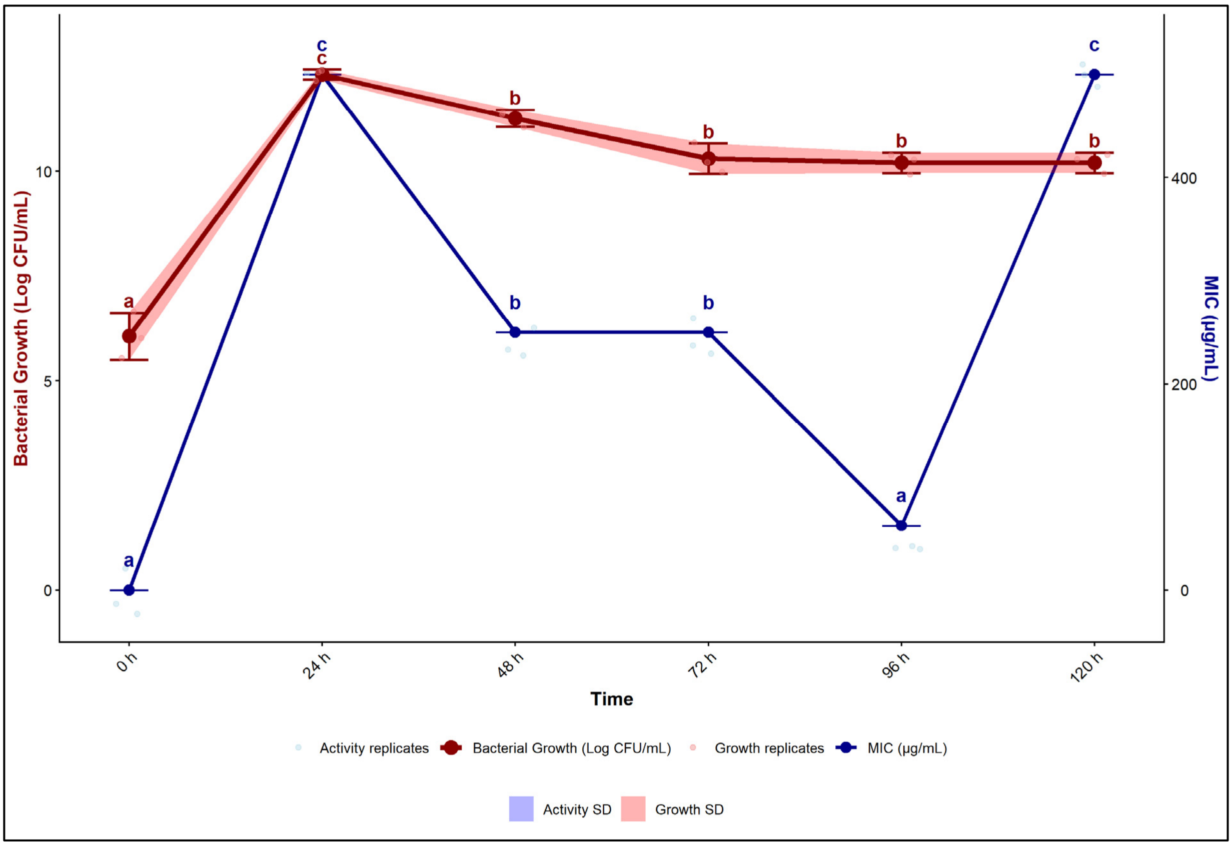

Figure S1. Growth kinetics of *Bacillus velezensis* B38 and corresponding antibacterial activity against *Escherichia coli* 18/22 in medium supplemented with 2% potato peel waste (PPW). Data are expressed as mean ± standard deviation (n = 3). Different lowercase letters indicate significant differences (Tukey's HSD test, $P < 0.05$).

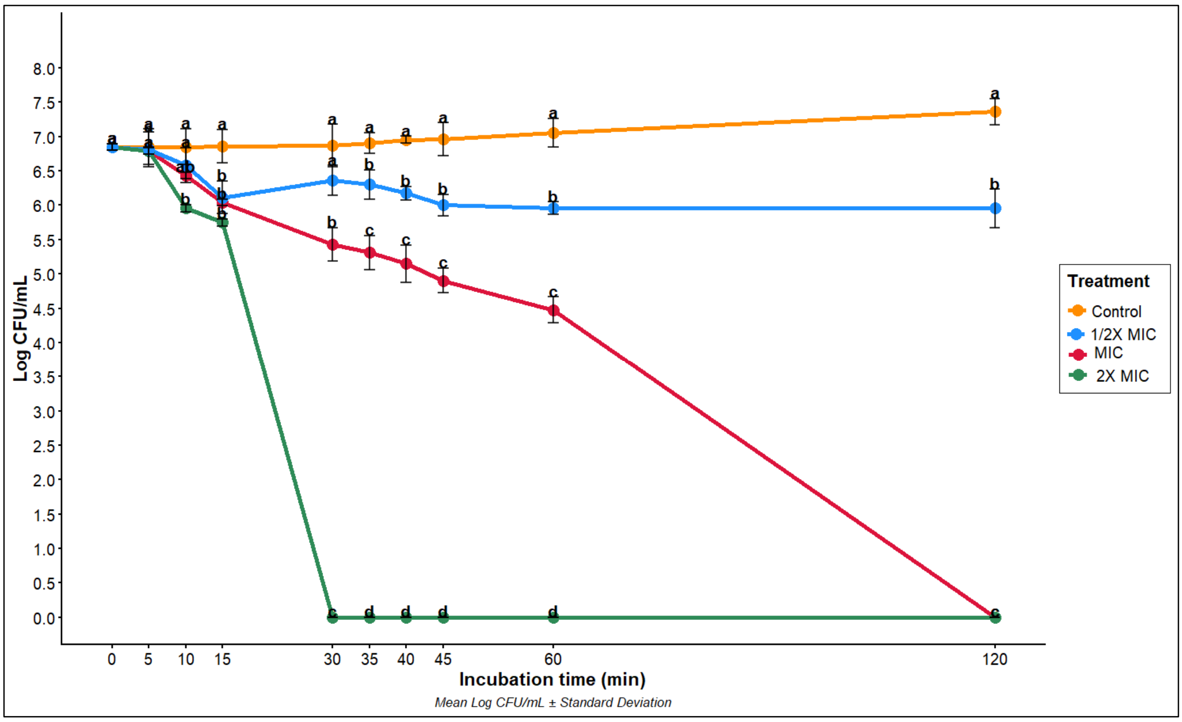

Figure S2. Time-kill kinetics of ethyl acetate extract obtained from *Bacillus velezensis* B38 cultivated with 2% PPW against *Escherichia coli* 18/22.

Data are expressed as mean ± standard deviation (n = 3). Different lowercase letters indicate statistically significant differences among treatments at each time point (Tukey's HSD test, $P < 0.05$).

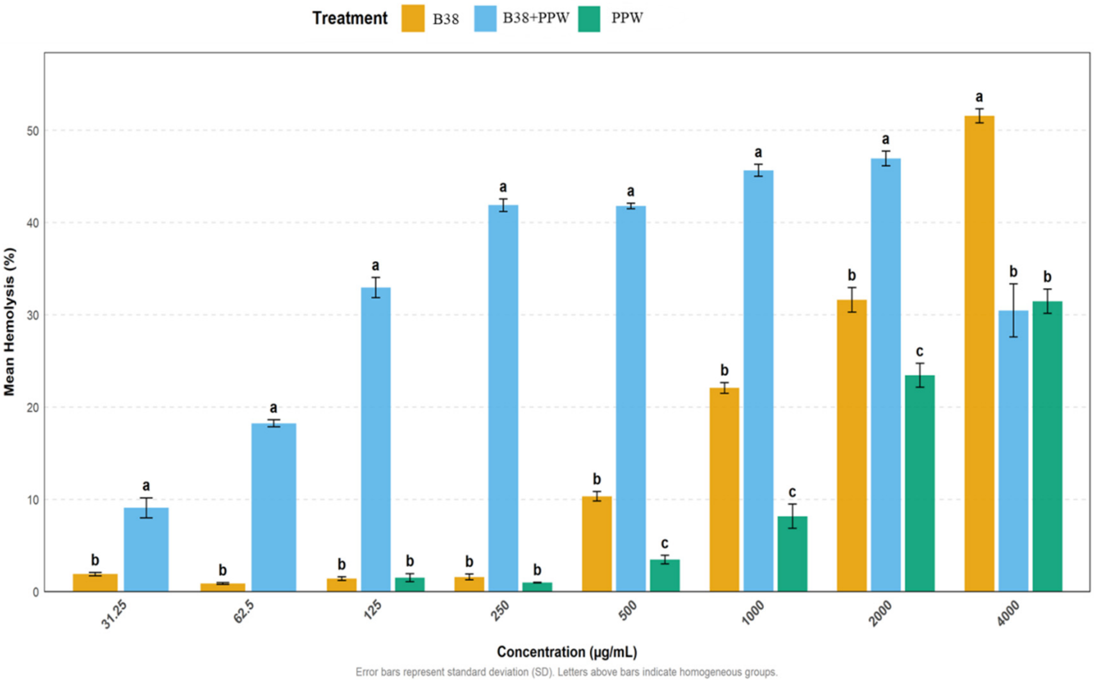

Figure S3. The hemolytic activity against human erythrocytes. Hemolytic activity (%) of B38, B38+PPW, and PPW extracts at increasing concentrations (31.25–4000 µg/mL).

Data are expressed as mean ± standard deviation (n = 3). Different lowercase letters above bars indicate statistically significant differences among treatments at each concentration (one-way ANOVA followed by Tukey's HSD post-hoc test, $p < 0.05$)

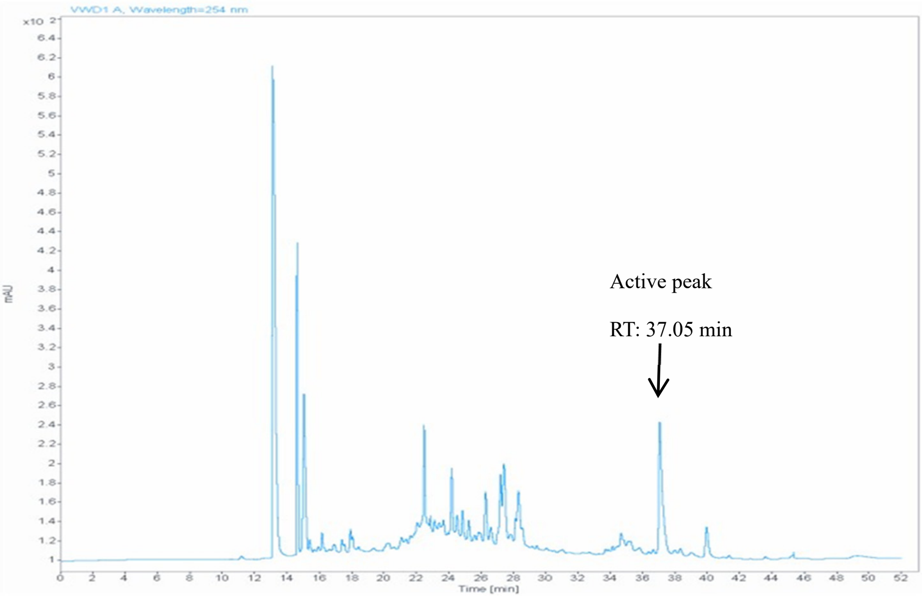

Figure S4. HPLC profile of the active fraction at 254 nm.

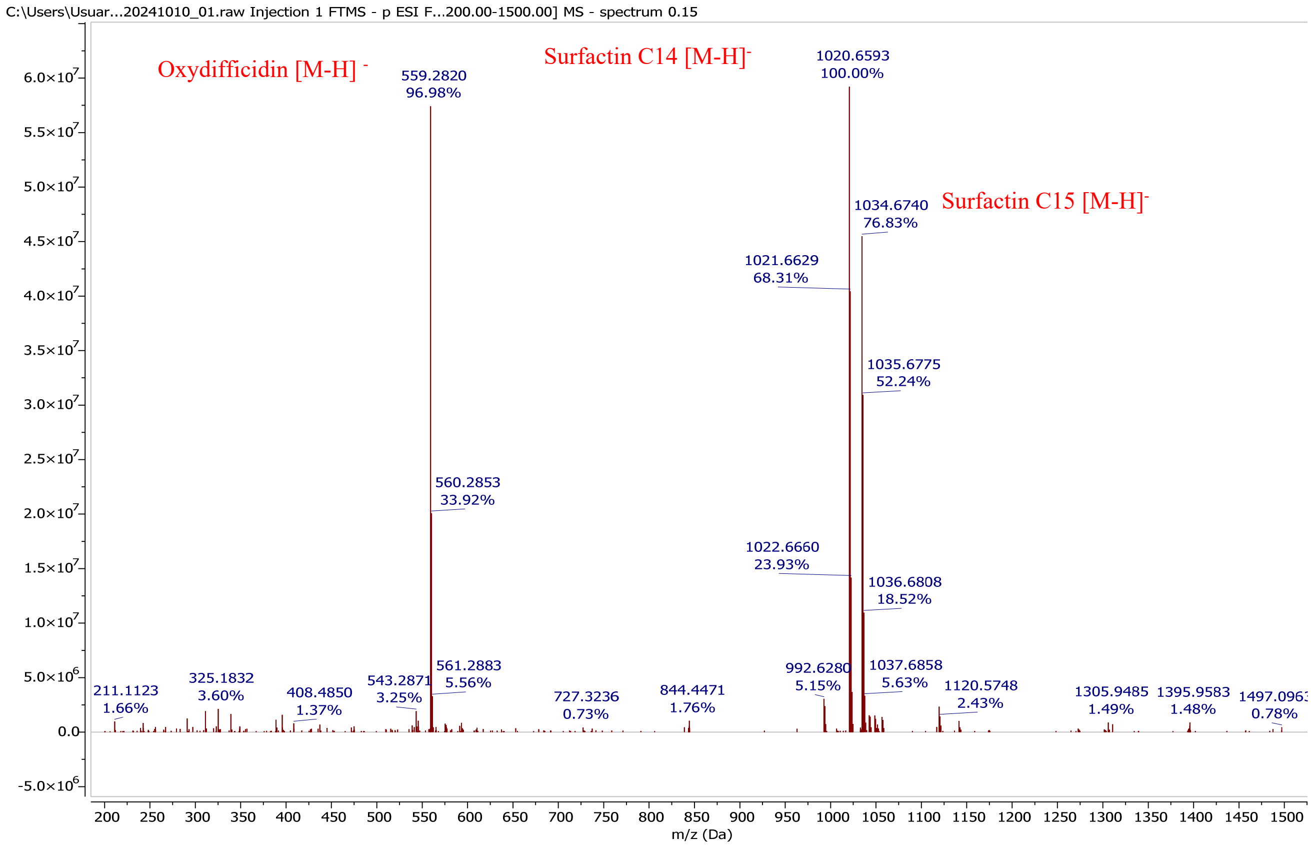

Figure S5. (-) HR-ESI mass spectrum of the active peak.

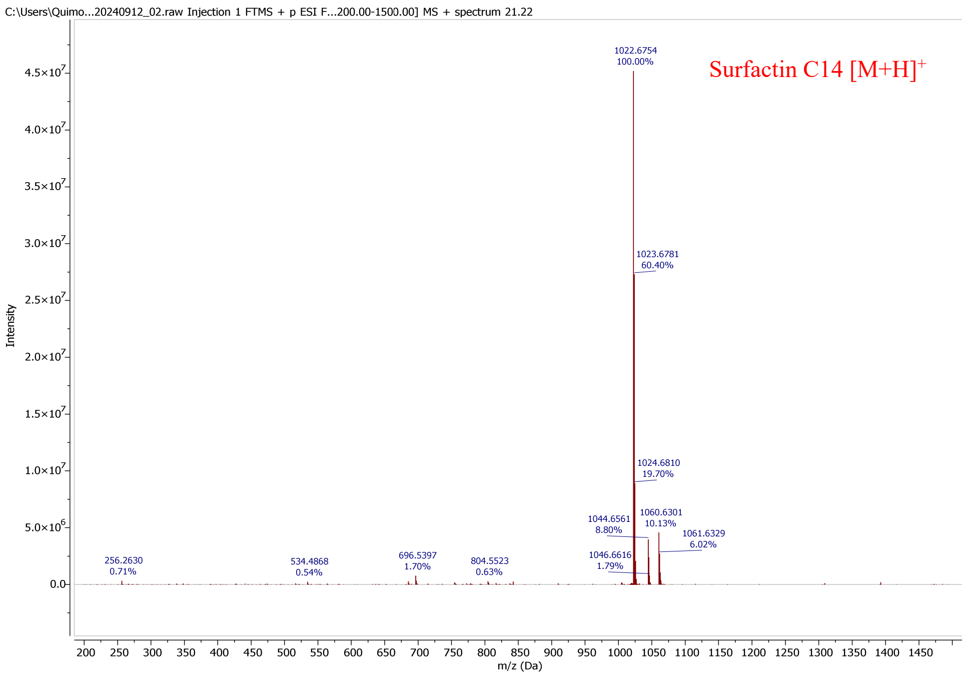

Figure S6. (+)-HRESIMS of surfactin C14.

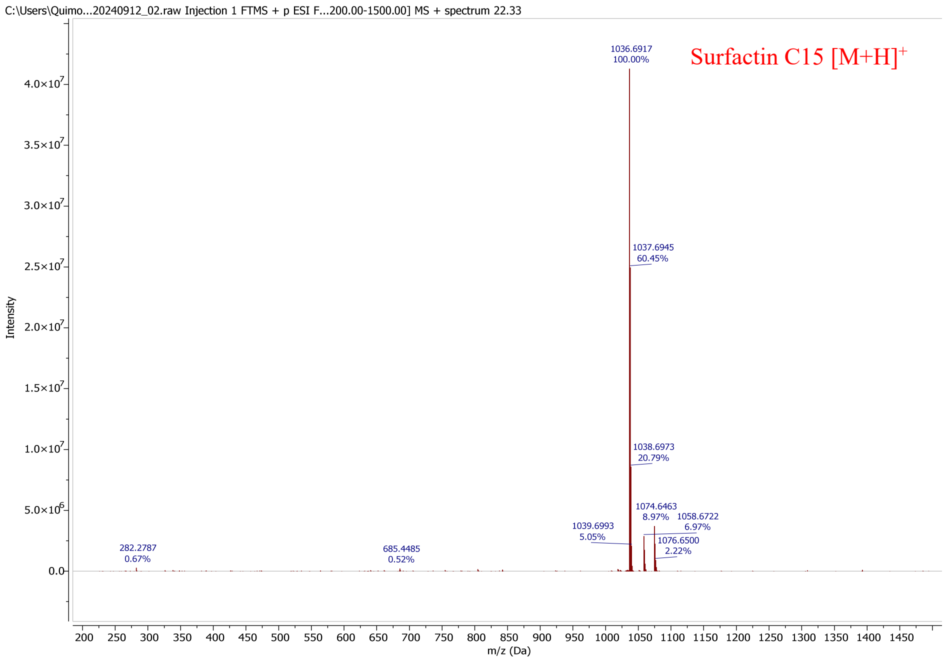

Figure S7. (+)-HRESIMS of surfactin C15.

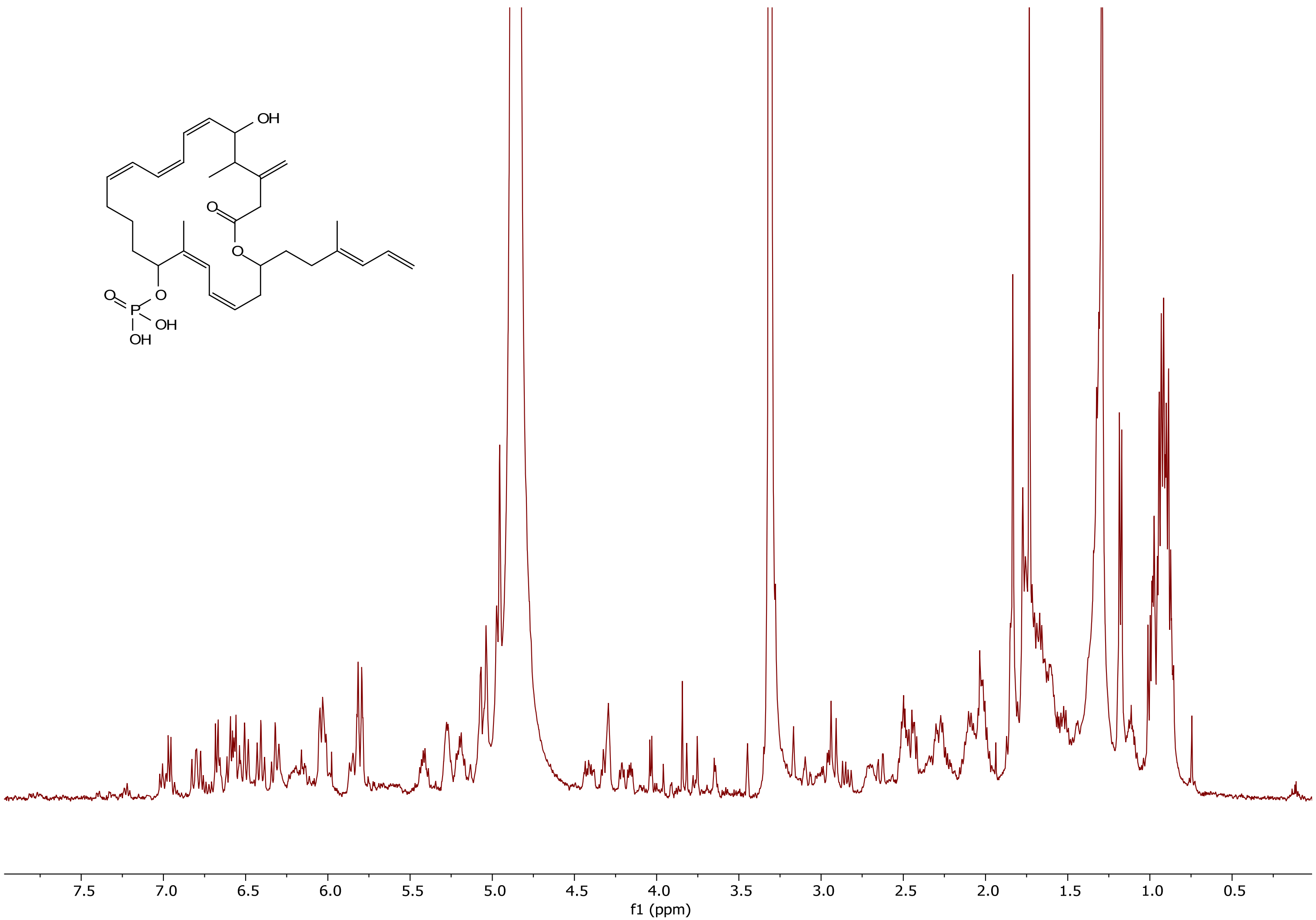

Figure S8. ^{1}H NMR spectrum (500 MHz, CD_3OD) of oxydifficidin.

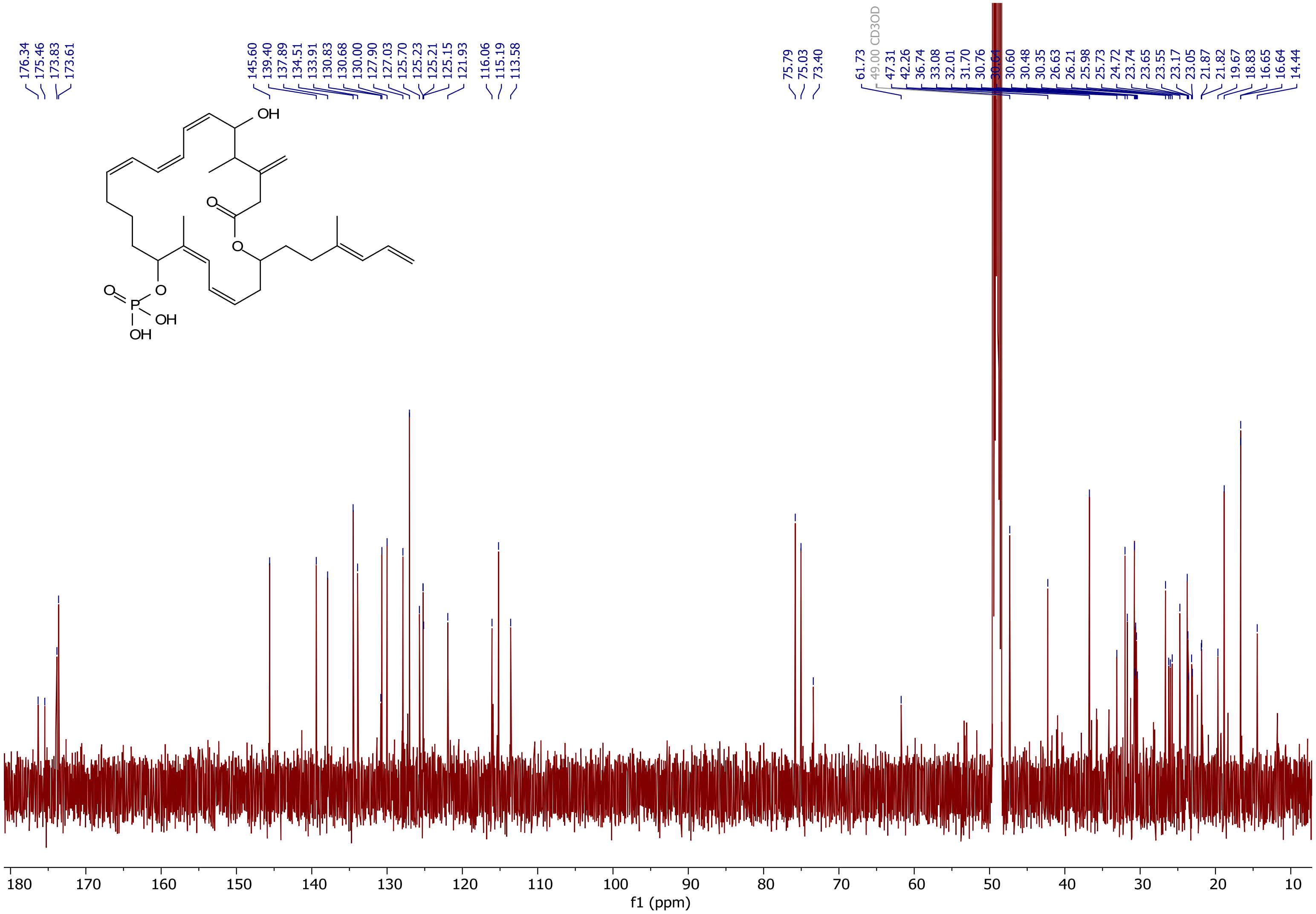

Figure S9. ^{13}C NMR spectrum (125 MHz, CD_3OD) of oxydifficidin.

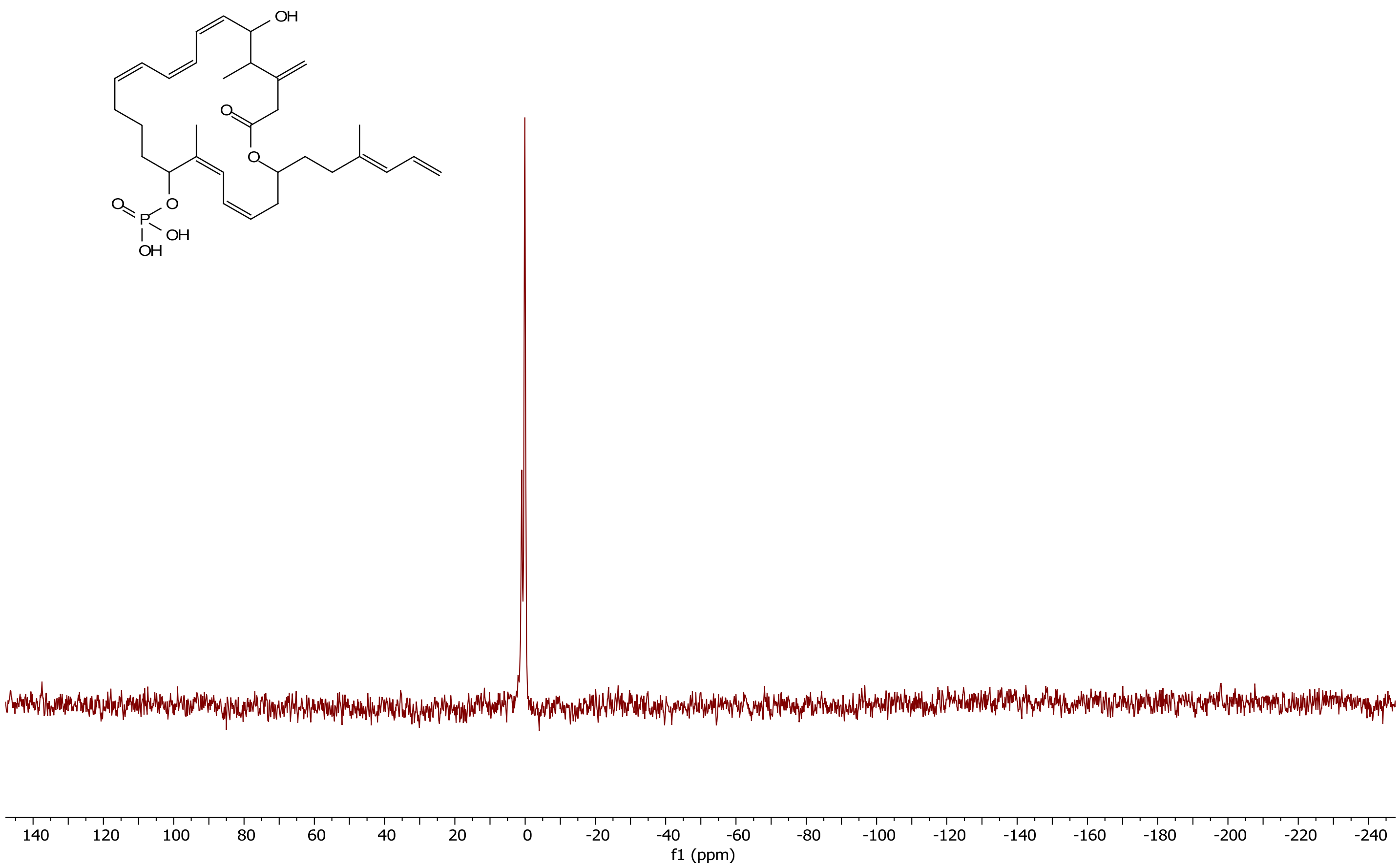

Figure S10. ^{31}P NMR spectrum of oxydifficidin.

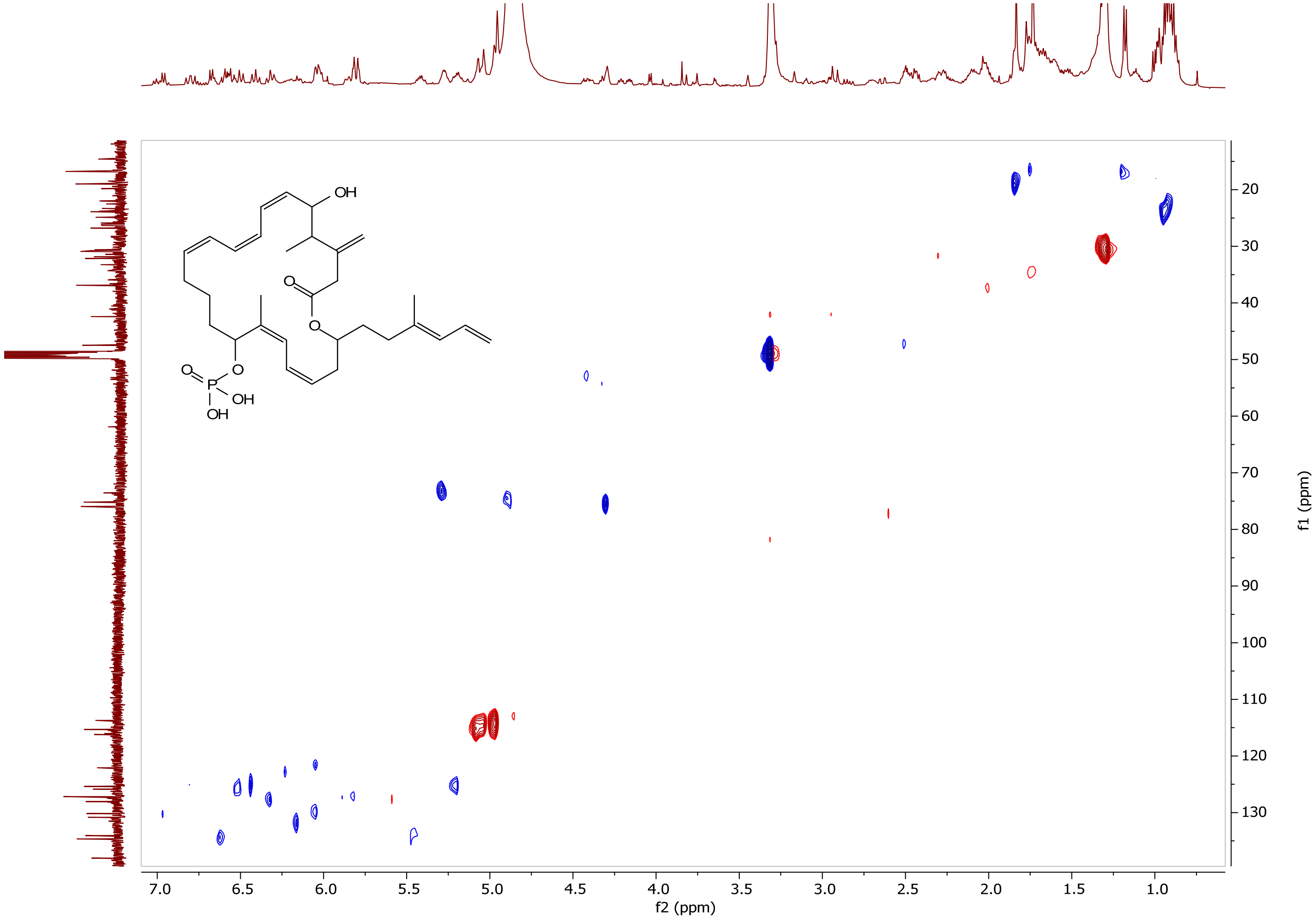

Figure S11. HSQC spectrum of oxydifficidin.

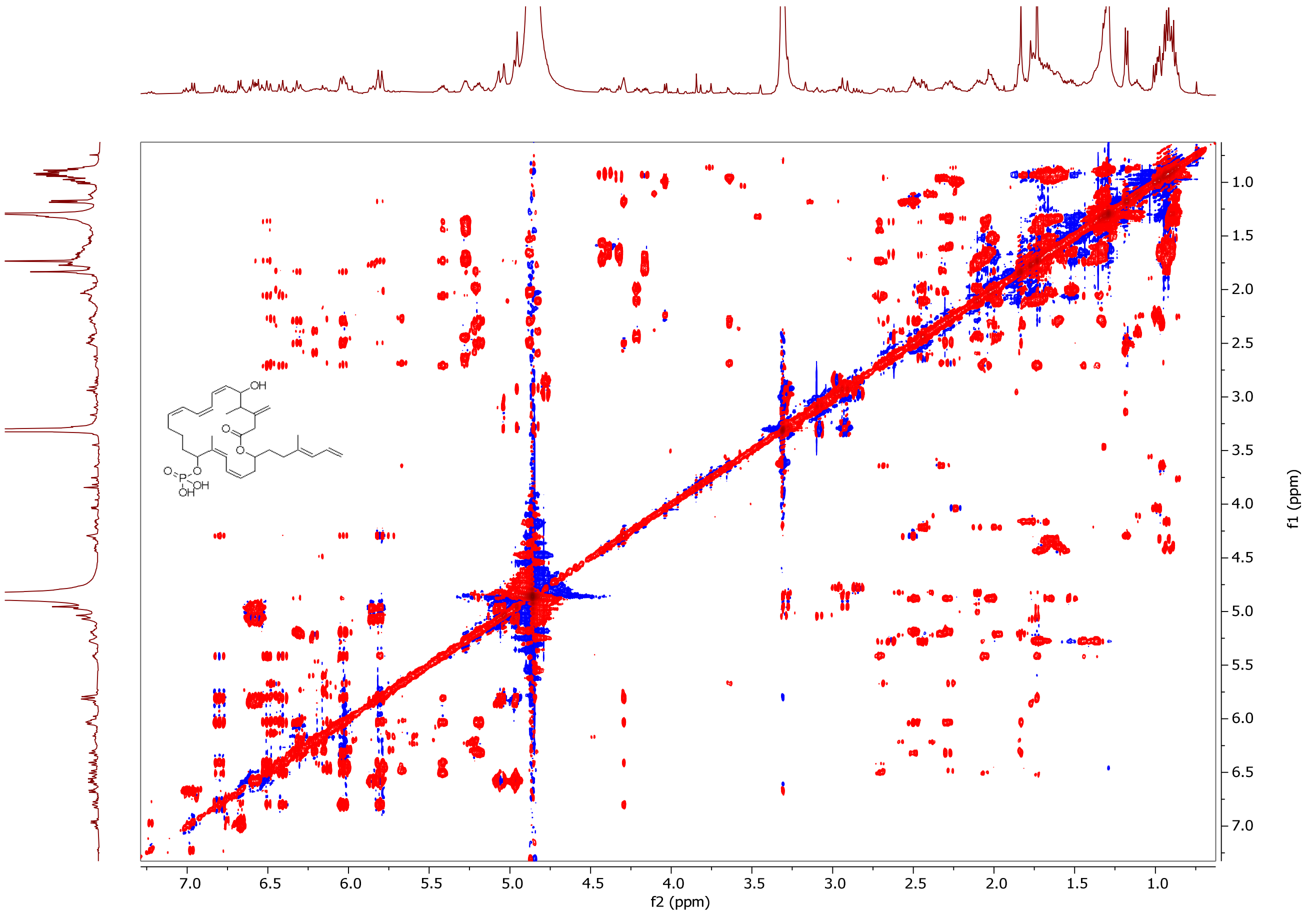

Figure S12. TOCSY spectrum of oxydifficidin.

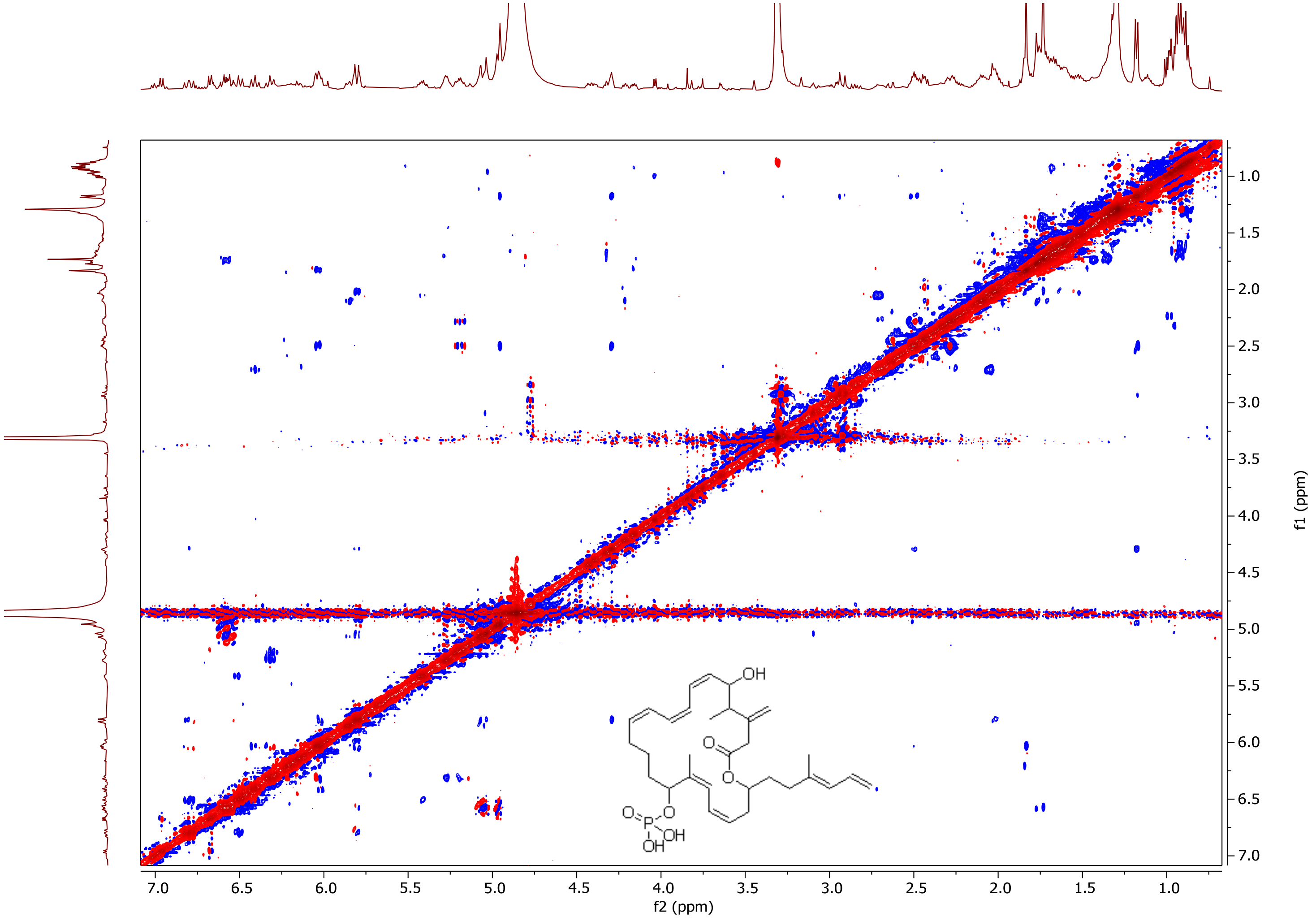

Figure S13. NOESY spectrum of oxydifficidin.

Table S1. Composition of potato peel (PPW) powder.

Composition	g/100g DW
Total polyphenols	0.429
Flavonoids	0.07
Carbohydrate content	28.58
Nitrogen content	1.552
Ash content	9.365
Moisture	15.95

Table S2. Partial purification of antibacterial agents from *Bacillus velezensis* B38 cultured in 2% PPW.

Extracts	Antibacterial activity against *E. coli* 18/22 MIC (µg/mL)
Cell Free Supernatant	62.5 µg/mL
EtOH extract	62.5 µg/mL
Hexane fraction	NA
Dichloromethane fraction	500 µg/mL
Ethyl acetate fraction	125 µg/ml
BuOH fraction	1000 µg/mL

Table S3. Physico-chemical characterization of the ethyl acetate active fraction of *Bacillus velezensis* B38 cultured in 2% PPW.

Physic-chemical treatments	Residual activity (%)
**Temperature**	
40°C, 60°C, 80°C	100%
100°C	25%
**pH**	
4, 6, 8, 10, 12	100%
**Treatment with enzymes**	
Pepsin, Chymotrypsin, pepsin and chymotrypsin	100%

Table S4. NMR data of oxydifficidin, 500 MHz, CD_3OD

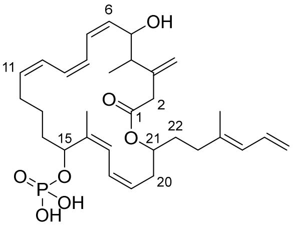

	Reported data[1]		Experimental data	
Position	δ_H (m, *J*, Hz)	δ_C (m)	δ_H	δ_C
1		174.0, C		174.1
2	3.29 (m)<br>2.92 (d, 15.6, 6.0 Hz)	42.4, CH_2	3.22/ 2.93	42.3
3		145.2, C		145.2
4	2.50 (m)	47.4, CH	2.50	47.2
5	4.30 (brt, 3.5)	75.8, CH	4.31	75.7
6	5.80 (dd, 12.0, 4.9)	137.3, CH	5.80	137.3
7	6.80 (dd, 14.9, 11.6)	125.3, CH	6.75	125.4
8	6.03 (m)	130.0, CH	6.02	129.3
9	6.32 (t, 11.2)	127.2, CH	6.34	125.4
10	6.41 (t, 11.2)	127.3, CH	6.47	125.4
11	5.44 (dt, 10.0, 10.0, 5,4)	134.1, CH	5.41	134.2
12	2.64 (m)<br>2.04 (m)	26.7, CH_2	2.64/ 2.04	26.7
13	1.77 (m)<br>1.34 (m)	24.2, CH_2	1.67/ 1.32	24.8
14	1.72 (m)<br>1.43 (m)	34.4, CH_2	1.71/ 1.42	35.2
15	5.27 (m)	73.6, CH	5.22	73.1
16		141.5, C	1.81	141.8
17	6.64 (m)	122.1, CH	6.02	121.7
18	6.63 (t, 11.2)	128.0, CH	6.33	128.0
19	5.21 (m)	125.3, CH	5.16	124.8
20	2.52 (m)<br>2.33 (m)	31.6, CH_2	2.51/ 2.25	31.5
21	4.90 (m)	75.2, CH	4.86	75.2
22	1.67 (m)<br>1.50 (m)	31.9, CH_2	1.63/ 1.50	31.7
23	2.00 (m)	37.7, CH_2	2.00	36.6
24		139.5, C		139.2
25	5.79 (d, 8.2)	127.0, CH	5.77	126.9
26	6.61	134.5, CH	6.56	134.5
27	5.05 (dd, 16.9, 7.7)<br>4.98	115.3, CH_2	5.05/ 4.97	115.5
28	1.73, (s)	16.8, CH_3	1.72	16.7
29	4.95 (brs), 4.86	113.7, CH_2	4.97/ 4.84	113.9
30	1.17 (d, 7.2)	16.7, CH_3	1.16	16.5
31	1.83, (s)	19.0, CH_3	1.84	19.7

[1]Kan Jingbo, Morales Adrian, Hernandez Yozen, Ternei Melinda A, Lemetre Christophe, Maclntyre Logan W, Biais Nicolas, Brady Sean F (2024) Oxydifficidin, a potent *Neisseria gonorrhoeae* antibiotic due to DedA assisted uptake and ribosomal protein RplL sensitivity eLife 13:RP99281. https://doi.org/10.7554/eLife.99281.3.

Table S5. Resistance profile of *Escherichia coli* 18/22 strain.

Tested antibiotics (abbreviation, the amount of each antibiotic/disk)	Diameter of inhibition zone (mm)	Interpretation
Ciprofloxacin (CIP) 5 µg	0	R
Ofloxacin (OFX) 5 µg	0	R
Nalidixic acid (NAL) 30 µg	0	R
Sulfamethoxazole and Trimethoprim (SXT) 25 µg	0	R
Gentamicin (GMN) 10 µg	8	R
Tobramycin (TMN) 10 µg	8	R
Cefixime (FIX) 5 µg/disc	10	R
Amikacin (AKN) 30 µg	18	S
Nitrofurantoin (NFE) 300 µg	21	S
Fosfomycin (FOS) 200 µg	32	S

The diameter of the inhibition zones were interpreted according to the Clinical and Laboratory Standards Institute M100-S26 (2016) and the examined isolate was reported as "R" resistant, "I" intermediate, "S" sensitive

Table S6. Oxydifficidin, surfactin C14, surfactin C15, and ciprofloxacin characteristics and structures obtained from PubChem.

Sample name	oxydifficidin	Surfactin C14	Surfactin C15	Ciprofloxacin
**CID**	196326	44227776	44227775	2764
**Molecular formula**	$C_{31}H_{45}O_7P$	$C_{52}H_{91}N_7O_{13}$	$C_{53}H_{93}N_7O_{13}$	$C_{17}H_{18}FN_3O_3$

Table S7. Grid box parameters of target ligands: the stx2 subunit A and AcrB of the AcrAB-TolC efflux pumps.

Receptor	amino acids of the active site	Grid Box Center Coordinates			Grid Box Size
		x	y	z	
Stx2 subunit A	Tyr77, Tyr114, Glu167, Arg170, Trp203 et Arg205	-0.239	63.27	8.441	30 x 30 x 44
AcrB	Ser46, Gln89, Glu130, Ser135, Phe136, Val139, Gln176, Lys292, Tyr327, Val571, Arg620, Phe628, Gln151, Phe178, Gly179, Ser180, Asn274, Asp276, Ile277, Ala279, Ser287, Pro326, Phe610, Val612, Phe615	34.807	-57.437	-46.428	44 x 44 x44
